# Advanced sampling simulations of coupled folding and binding of phage P22 N-peptide to boxB RNA

**DOI:** 10.1016/j.bpj.2024.08.022

**Published:** 2024-08-28

**Authors:** Luis Vollmers, Martin Zacharias

**Affiliations:** 1 Physics Department and Center of Protein Assemblies, Technical University Munich, Garching, Germany

## Abstract

Protein-RNA interactions are crucially important for numerous cellular processes and often involve coupled folding and binding of peptide segments upon association. The Nut-utilization site (N)-protein of bacteriophages contains an N-terminal arginine-rich motif that undergoes such a folding transition upon binding to the boxB RNA hairpin loop target structure. Molecular dynamics free energy simulations were used to calculate the absolute binding free energy of the N-peptide of bacteriophage P22 in complex with the boxB RNA hairpin motif at different salt concentrations and using two different water force field models. We obtained good agreement with experiment also at different salt concentrations for the TIP4P-D water model that has a stabilizing effect on unfolded protein structures. It allowed us to estimate the free energy contribution resulting from restricting the molecules’ spatial and conformational freedom upon binding, which makes a large opposing contribution to binding. In a second set of umbrella sampling simulations to dissociate/associate the complex along a separation coordinate, we analyzed the onset of preorientation of the N-peptide and onset of structure formation relative to the RNA and its dependence on the salt concentration. Peptide orientation and conformational transitions are significantly coupled to the first contact formation between peptide and RNA. The initial contacts are mostly formed between peptide residues and the boxB hairpin loop nucleotides. A complete transition to an α-helical bound peptide conformation occurs only at a late stage of the binding process a few angstroms before the complexed state has been reached. However, the N-peptide orients also at distances beyond the contact distance such that the sizable positive charge points toward the RNA’s center-of-mass. Our result may have important implications for understanding protein- and peptide-RNA complex formation frequently involving coupled folding and association processes.

## Significance

The coupled folding and binding of protein segments upon association with RNA is frequently found during protein-RNA complex formation. Molecular dynamics free energy simulations have been used to investigate the specific association of a positively charged peptide that forms an α-helical structure in complex with an RNA hairpin structure. The calculations allowed us to estimate the contributions of peptide folding to binding and how it depends on salt concentration and force field model for water. The study gives also insights into how orientation and folding of the peptide evolve upon association/dissociation of the peptide relative to RNA.

## Introduction

The binding of proteins to RNA is often mediated by short positively charged and largely unfolded peptide segments that adopt a folded conformation only in the complexed state (1). Such segments are, for example, found in proteins that are part of the ribosome or the splicing machinery of eukaryotes but also in proteins of viruses or bacteriophages that interact with viral or host RNAs (2,3,4). Upon coupled folding and binding the mostly positively charged residues on the peptide segment adopt placements that optimize the interaction with the electrostatic field of the RNA (1,5). It involves interactions of the basic amino acids with the sugar-phosphate backbone, which is a major part of most protein-RNA contacts. In addition, also hydrogen bonds are formed with nucleobases and additional hydrophobic and van der Waals contacts. Numerous structures of protein-RNA complexes have been solved in recent years either as part of larger multicomponent assemblies (e.g., ribosome structures, splicing machinery) (6,7,8) or as isolated peptide-RNA complexes (3). However, the mechanism of coupled folding and binding of proteins/peptides to the binding sites on the RNA molecules is much less well studied and understood. It includes questions about the specific initial contacts between the partners, intermediate states, and if an orientation and folding process is already initiated before contacts are formed (9,10,11). This latter mechanism may play a role due to the strong electrostatic field of the RNA and the often positive charge of the peptide binding segment.

A narrow major groove and a flat minor groove are characteristic for the canonical A-form of double-stranded RNA and therefore do not offer cavities or clefts for specific peptide binding (1,12,13). Thus, most protein-RNA binding involves noncanonical RNA structures such as internal loops or extra bulge nucleotides that widen the major groove (12,13). Important recognition sites on folded RNA molecules are hairpin loop elements that reoccur in many folded RNA structures. The loop formation results in a widening of the major groove near hairpin loops such that peptides even in an α-helical conformation can easily fit and can form many close contacts to the RNA backbone and the nucleobases (14).

**Figure 1:**
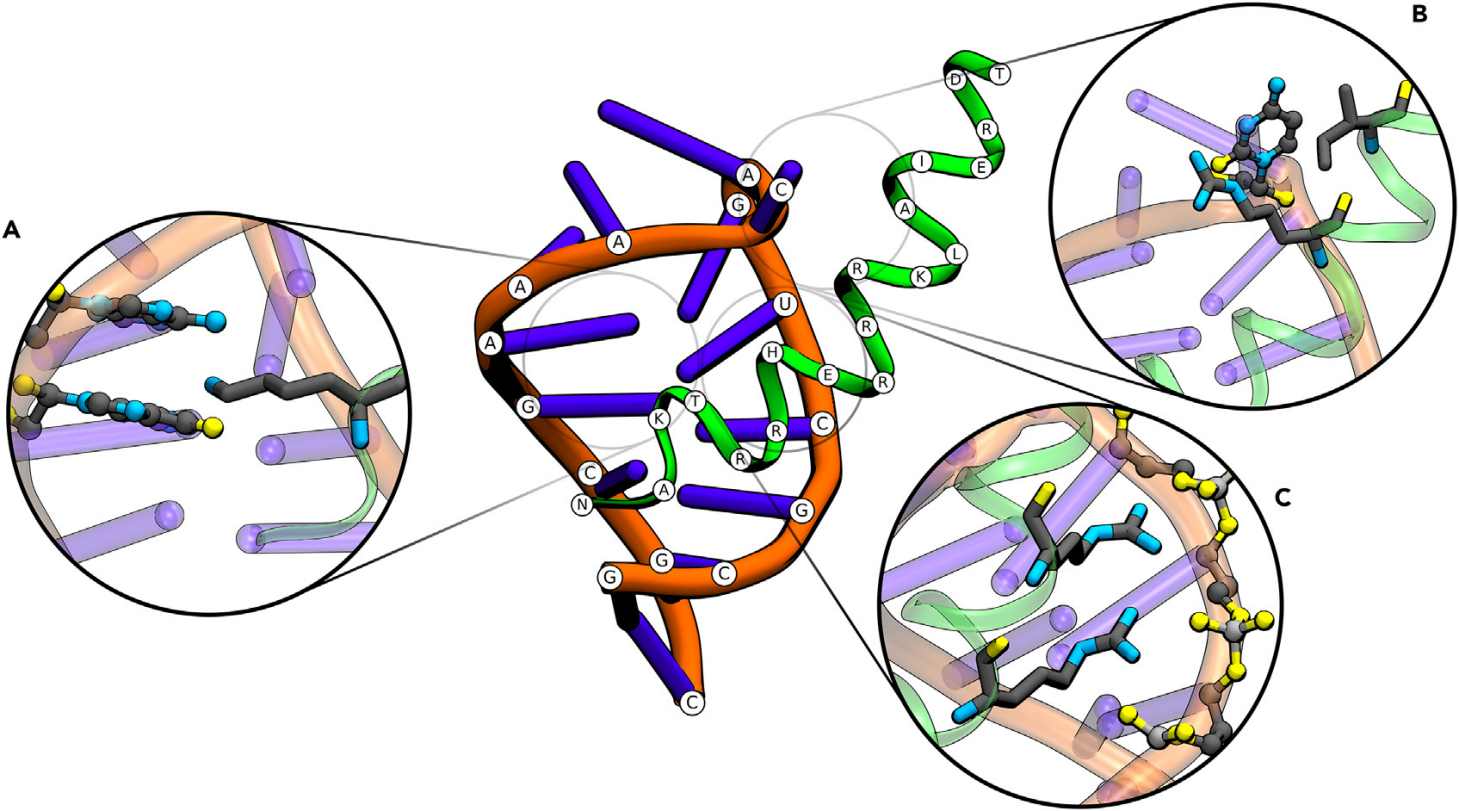
Structure of the 19 residue bacteriophage P22 N-peptide (*green cartoon*, sequence: NAKTRRHERRRKLAIERDT) in complex with the 15-mer boxB RNA hairpin motif (*orange* and *purple*, sequence: 3′-GCGCUGACAAAGCGC) (15). The three main contact areas are illustrated in three separate panels, with (*a*) indicating the K3 contacting the guanine (G6) and adenine (A7) nucleobases, (*b*) showing loop base C8 contacting one arginine residue and an isoleucine residue, and (*c*) capturing the two most prominent arginine contacts to the phosphate backbone. For clarity hydrogen atoms are omitted. Peptide atoms are in licorice and RNA atoms in CPK representation (PDB: 1A4T (15)).

For example, the Nut-utilization protein (N-protein) of bacteriophages contains a basic (arginine-rich) conserved N-peptide segment, which undergoes a folding transition to form a bent α helix upon binding to the boxB RNA hairpin structure. The nacent complex of N-protein and the boxB element leads to association with RNA polymerase forming an antitermination complex (15) that prevents premature transcription termination (16). It plays an important role in gene regulation and phage replication regulation (17). The binding of positively charged peptide segments to hairpin and internal loops is also found in several other viral systems including the tat-TAR interaction and rev-RNA interaction in HIV (10,12,18,19). The structure of the N-peptide segment of phage P22 bound to the boxB has been determined using NMR spectroscopy (15) (see Fig. 1) and has been extensively investigated using biochemical and biophysical approaches (10,20,21,22). The N-peptide consists of 19 amino acids (including 9 basic residues) (23), which bind to the widened major groove of the boxB RNA hairpin loop (15). Due to its relatively small size, the P22 N-peptide-boxB complex forms a well-suited model system for computational studies. A closely related system from bacteriophage λ has been studied using the nonlinear Poisson-Boltzmann continuum solvent model to analyze the salt dependence of the binding process in good agreement with experiment (24). It has also been studied using the molecular mechanics Poisson-Boltzmann approach aiming to analyze energetic contributions to binding and to explain the contribution of each individual side chain on the binding affinity (10). The study also indicated that the RNA electrostatic field may have an influence on preorienting and on the peptide folding process close to the RNA but before contact formation. However, these studies considered only a simplified implicit model of the surrounding aqueous solution and, secondly, focus mostly on the end-state, the bound complex structure.

In this study we use binding free energy molecular dynamics (MD) simulations to study the peptide-RNA binding process in atomic detail with an explicit representation of the surrounding water molecules and ions. In a first set of simulations we investigate the dissociation/association including geometric and conformational restraints on binding partners based on an approach by Woo and Roux (25). The inclusion of such restraints allows rapid convergence of the calculated absolute binding free energy and also yields results on free energy contributions to the binding process. One of the used water models closely reproduces the experimental binding free energy validating the force field approach. It also allows us to estimate the free energy contribution of orienting and folding of the peptide relative to the boxB RNA partner during coupled folding and binding.

In separate simulations the dissociation/association process was induced along a plain center-of-mass distance coordinate between peptide and RNA binding region. In contrast to the simulations including geometric restraints, the peptide has full freedom with respect to conformation and orientation relative to the RNA during such binding or unbinding process. For this case it is much more difficult to achieve converged free energy results but it allows us to follow the conformational and orientational changes of the N-peptide, and to study the onset of folding or reorientation along the distance coordinate relative to the RNA partner. The simulation studies were performed using different salt concentrations and also two different water models. One water model, TIP3P (26,27), is known to stabilize secondary structures in proteins, whereas the other selected water model, TIP4P-D (28,29,30) has the tendency to destabilize folded protein structures and to be better suited for applications that involve disordered peptides (31). We find that loss of direct contacts between peptide and RNA results in rapid loss of orientational and conformational correlations. Hence, initial contacts are decisive for the formation of intermediates and the subsequent steps of the binding process. The effects of selected order parameters, influence of the water model, and added ions are also discussed.

## Methods

### Simulation setup and MD simulations

The Amber program package (32,33) was used to conduct all simulations and parts of the analysis. The peptide-RNA complex (PDB: 1A4T (15)) was solvated in an octahedral box using either the TIP3P water model (26) or the TIP4P-D model (29). Sodium and chloride ions were added to neutralize the system and to achieve a final ion concentration of 81, 177, and 450 mM, which corresponds approximately to the range of ion concentrations observed in bacteria. After extensive energy minimization (2000 steps) the systems were gradually heated in steps of 100 K up to a target temperature of 300 K within 4 ns. During this phase, all nonhydrogen atoms of peptide and RNA were positionally restrained to the start structure (12 kcal mol^−1^ Å^−2^). The positional restraints were gradually released within additional 12 ns simulation time at 300 K and under constant pressure of 1 bar. The nonbonded interactions were calculated by using the PME method with 9.0 Å real space cutoff (34), SHAKE constraints (35) are applied to hydrogen bonds, and an H-mass repartitioning (36) was conducted to enable a time step of 4 fs. The ff14SB protein force field (37) was used and the OL3 force field for RNA (38). For equilibration simulations the Berendsen coupling scheme was used to control temperature and pressure (39) and during production runs a stochastic coupling was employed (40,41).

### Replica-exchange and conventional umbrella sampling simulations along a peptide-RNA separation coordinate

The dissociation/association of the peptide-RNA complex was induced using a distance reaction coordinate between the centers-of-mass of the peptide and the RNA. As initial structure the equilibrated complex from the unrestrained simulations was used. The starting structures for umbrella sampling (US) (42,43) were generated by a set of consecutive MD simulations including a quadratic penalty potential at 1 Å steps along the center-of-mass distance with a force constant of 0.5 kcal mol^−1^ Å^−2^ for a distance range of 8–25 Å and in 2 Å steps for 26–40 Å distances. The simulations in each window were extended to 250 ns and using a force constant of 2.0 kcal mol^−1^ Å^−2^. For the larger center-of-mass distances of 26–40 Å additional US simulations with 150 ns per window and force constants of 1.0 and 0.5 kcal mol^−1^ Å^−2^, respectively, were performed. To improve the sampling in the smaller distance regime we also performed Hamiltonian replica-exchange US (HREUS) (44,45) simulations in the distance range of 8–25 Å (force constant: 0.5 kcal mol^−1^ Å^−2^ and exchange attempts every 1 ps). The acceptance rate was at least 0.1 for all exchange attempts. The free energy curve was obtained via the weighted histogram analysis method (46,47). The standard deviation and error were estimated from block averaging each umbrella window with a block count of 10.

### Trajectory analysis and onset peptide folding

The Python package MDAnalysis (48,49) was used to conduct a close contact analysis, a secondary structure analysis and an orientational analysis of the peptide relative to boxB RNA. The close contact analysis includes the average closest contact distance $\left\langle r_{\text{cc}}\right\rangle$ and the frequency of a contact closer than 4 Å, $f_{\text{cc}}$. $\left\langle r_{\text{cc}}\right\rangle$ is calculated with respect to the time frames t of an umbrella window i.

(1) $${\left\langle r_{\text{cc}}\right\rangle }_{i}=\frac{1}{N_{t}}\sum_{t}r_{\text{cc}}\left(t\right)$$

The standard deviation (50) of this metric, $\delta \left\langle r_{\text{cc}}\right\rangle$, serves as an additional measure of early contact formation. $f_{\text{cc}}$ is also calculated with respect to the trajectory frames t of the ith umbrella window. $f_{\text{cc}}$ assumes values between 0 (no close contact frames) and 1 (every frame with close contacts).

(2) $$f_{i,\text{cc}}=\frac{1}{N_{t}}\sum_{t}[r_{\text{cc}}\left(t\right)\leq 4\;Å]$$

As a quantification of peptide prefolding the angle ω between the peptide-RNA center-of-mass vector and several structurally significant peptide vectors was calculated. Structural peptide vectors include the principal axis ${\vec{I}}_{1}$, the dipole moment $\vec{d}$ and the end-to-end vector ${\vec{R}}_{\;\text{ee}}$ resulting in three distinct angle spaces $\Omega^{I_{1}}$, $\Omega^{d}$, and $\Omega^{\text{ee}}$. Furthermore, from a bulk simulation, a reference angle distribution $P_{\;\text{ref}}\left(\omega \right)$ is computed between these vectors and the coordinate system’s *x* axis. Finally, the total variation distance $\left(D_{\;\text{TV}}\right)$ is calculated between measurement and reference for the discretized angle space Ω(51).

(3) $$D_{\text{TV}}\left(\Omega \right)=\sum_{\omega \in \Omega }\left|P\left(\omega \right)-P_{\text{ref}}\left(\omega \right)\right|$$

Thus, $D_{\;\text{TV}}$ values approaching the minimum of 0 correspond to an unfettered peptide molecule, while high values approaching a maximum of 2 correspond to strong coupling between N-peptide and boxB RNA. By combining this measurement with the contact information, the order of binding and folding for the far center-of-mass distance regime can be deduced.

For association and binding, the helicity plays a vital role. Thus STRIDE (52) is used to measure the helicity of the N-peptide with respect to center-of-mass distance. The native contacts found in the PDB structure (15) are quantified using the MDAnalysis python package (48,49), which in turn follows the soft cutoff method described by Best et al. (53), resulting in a continuous, molecular measure $Q_{\text{nat}}$. Furthermore, hydrogen bonds are quantified via the MDAnalysis python package (48,49). The definition applies a 4 Å distance cutoff between donor and acceptor and 120° angle cutoff for the donor-hydrogen acceptor angle. The hydrogen bonds are counted residue-wise and are then averaged over the complete peptide, yielding ${\left\langle N_{\text{HB}}\right\rangle }_{\text{res}}$. The reason for measuring two different modes of intermolecular contacts stems from robustness efforts and the unique advantages each offers. Hydrogen bonds serve as reliable indicators of any binding progression. Conversely, the native contacts register progress specifically with respect to the PDB structure.

### Absolute binding free energies including geometrical and conformational restraints

**Figure 2:**
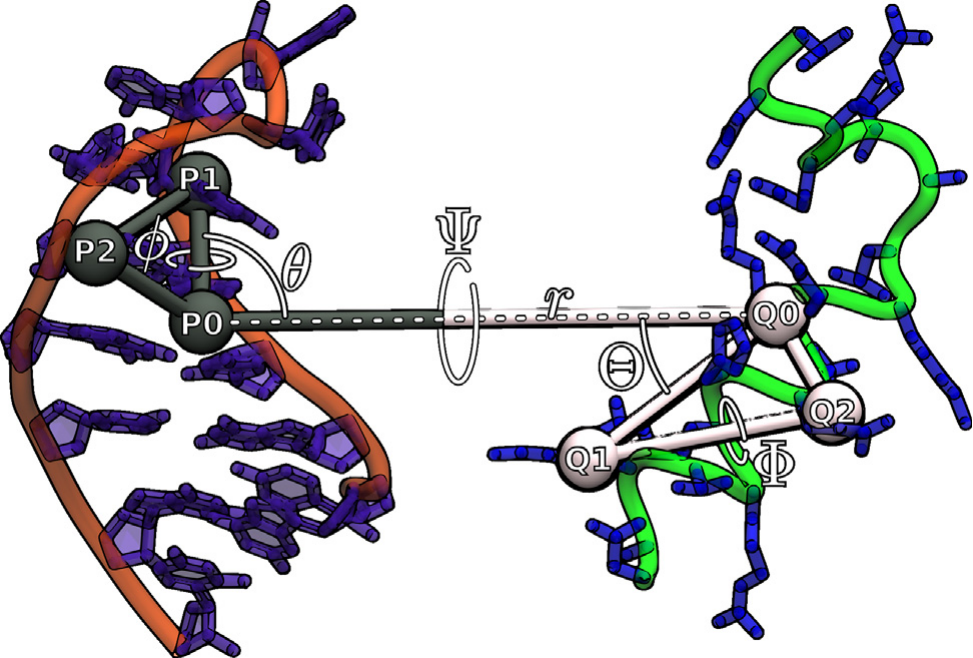
Illustration of three center-of-mass anchors and virtual bonds of different atom groups in boxB RNA (*orange*/*purple*, molecule; *black*, anchor beads) and the N-peptide (*green*/*blue*, molecule; *white*, anchor beads). The central anchor of each molecule, P0 and Q0, are defined as the centers-of-mass of all nonhydrogen atoms. P1 and P2 are defined as the centers-of-mass (nonhydrogen atoms) of residue G6 and A11, respectively. Q1 and Q2 are defined as the centers-of-mass (nonhydrogen atoms) of residue LYS3 and GLU8, respectively. The spherical angles of θ and ϕ are defined by the virtual bonds P1-P0-Q0 and P2-P1-P0-Q0. The orientational angles Ψ,Θ, and Φ are defined by the virtual bonds P1-P0-Q0-Q1, P0-Q0-Q1, and P0-Q0-Q1-Q2. The equilibrium angles for the axial and orientational restraining potentials are (in radians), $\Theta_{0}$ = 2.53, $\Phi_{0}$ = 2.7, $\Psi_{0}$ = −3.13, $\theta_{0}$ = 1.49, $\phi_{0}$ = −2.88, the force constant is in all cases, k= 100.0 kcal mol^−1^ rad^−2^.

Absolute binding free energies were calculated following a protocol implemented by Woo and Roux (25) (see also Boresch et al. (54)) in the following termed WR method. It includes axial and orientational restraints of the binding partners to define a direction of separation and to restrict the relative orientation of binding partners during a potential of mean force (PMF) calculation for partner separation. The associated quadratic restraining potentials are defined for virtual bonds between six anchor points, three within each binding partner. The anchor points represent the centers-of-mass of atom groups within each molecule (for the definitions see Fig. 2).

The reference axial and orientational angles were obtained from the equilibrium simulation of the complex. In addition, the conformation of binding partners is also restrained during the PMF calculation via a quadratic penalty function on the root mean-square deviation (RMSD) of only nonhydrogen atoms relative to the equilibrium complex. The free energy changes associated with the release of the restraints are only assessed at the endpoints of the separation PMF calculation, i.e., in the bound and fully separated states (bulk or unbound state). The inclusion of restraints allows a more rapid convergence of the PMF along the separation coordinate (55,56).

For the PMF calculation we used the HREUS technique in the distance range of 8–39 Å and exchange attempts every 1 ps. A force constant of 8.0 kcal mol^−1^ Å^−2^ was employed for 8–15 Å otherwise 4.0 kcal mol^−1^ Å^−2^. Simulations in each window were extended to 106–175 ns until convergence of the extracted distance histograms.

The free energy change associated with the release of the conformational restraints was calculated in an RMSD range of up to 15 Å with respect to the bound conformation for the N-peptide in the bulk using the HREUS technique. For the RNA in the bulk during HREUS simulations an RMSD range of up to 5.5 Å was employed. In the case of the conformational free energy simulations between 200 and 400 ns per HREUS window were required to achieve converged free energy results. A single step perturbation was sufficient for the RNA and the peptide at the complex site with the exemption of 450 mM system in TIP4P-D water. Here, the same procedure as for the bulk system was applied (quality control and the calculated free energy curves are provided in the supporting material, Figs. S1 and S2). The free energy changes associated with the release of the axial and orientational restraints were accomplished with a single-step perturbation using the Bennett acceptance ratio (BAR) method (57,58). For the evaluation of the conformational HREUS calculations the mBAR method was used (59) (with the pymbar package (59,60,61) and Scipy’s nquad function (62) in python (63,64,65,66)).

The setup, analysis, and quality control (67) of the WR method was automated for using the Amber package (available at github.com/Foly93/AmbeRouxWoo).

The absolute binding free energy is given by a summation of all restraint contributions (25,55). The contributions due to peptide and RNA restraints are denoted with subscripts p and n, and orientational and angular restraining contributions with o and a, respectively. Superscripts B and S denote the bulk and site contributions, respectively. The subscripts o and a can be further split into the Θ, Φ, Ψ, θ, and ϕ angle restraining contribution (see Fig. 2), $k_{B}$ is the Boltzmann constant and T is the simulation temperature. The PMF contribution along the separation distance contributes to $\Delta G_{b}^{⊖}$ via $-k_{B}T\;\ln\left(I^{*}S^{*}C\right)$.

(4) $$\Delta G_{b}^{⊖}=-k_{B}T\;\ln\left(I^{*}S^{*}C^{⊖}\right)+\Delta G_{p}^{B}+\Delta G_{n}^{B}+\Delta G_{o}^{B}-\Delta G_{p}^{S}-\Delta G_{n}^{S}-\Delta G_{o}^{S}-\Delta G_{a}^{S}$$

## Results

**Figure 3:**
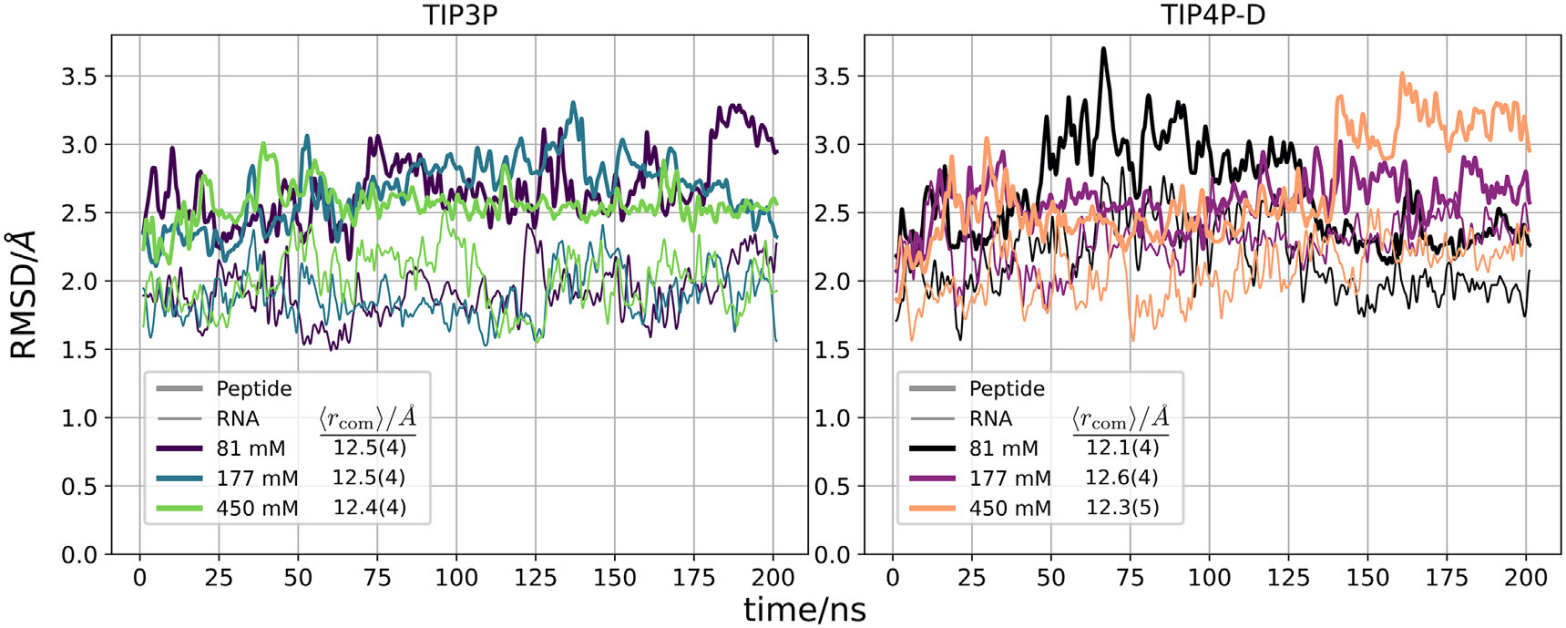
Calculated RMSD of only nonhydrogen atoms of the RNA (*thin line*) and peptide (*bold line*) with respect to the crystal structure during unrestrained MD simulations. Different line colors indicate results at three different salt concentrations (resulting in three RMSD curves for peptide and RNA, respectively). Note, a Gaussian filter with a window size of 20 was used to improve clarity. The average peptide-RNA center-of-mass distances at each ion concentration are also listed with their standard deviations in parentheses.

Conventional, unrestrained MD simulations of the N-peptide-boxB RNA complex were performed at three different salt concentrations (81, 177, and 450 mM NaCl) and using two different water models (TIP3P (27) and TIP4P-D (29)) to evaluate the conformational stability of the complex. The simulations were performed at 300 K and were extended to 200 ns. In all simulations the complex stayed relatively close to the start structure and no dissociation of the complex was observed with the center-of-mass distance between RNA and peptide remaining stable at ∼12.5 Å with standard deviation below 5% (see Fig. 3).

### Calculation of absolute binding free energies

The calculation of absolute binding free energies was performed following a protocol by Woo and Roux (25). The method includes geometrical and conformational restraints and includes the calculation of the free energy change along a directed distance coordinate. The free energy contributions from the restraints can be calculated in separate steps (see methods). The total (absolute) binding free energy is then a sum of contributions related to the restriction of relative orientation, the restriction of conformational freedom upon binding, and interactions along the distance reaction coordinate. The calculated binding free energy depends significantly on the water model and, as expected, also on the salt concentration (see Table 1). Using the TIP3P model yields consistently more favorable binding free energies by 6–7 kcal mol^−1^ compared with using the TIP4P-D water model. Note that the TIP4P-D model includes more favorable solute-solvent interactions by deliberately adjusting the Lennard-Jones parameters and is therefore expected to more strongly favor the unbound state than the TIP3P model (29).

**Table 1:** Table summarizing the contributions to $\Delta G_{b}^{⊖}$ obtained via the WR method

Contribution	TIP3P			TIP4P-D		
	81 mM	177 mM	450 mM	81 mM	177 mM	450 mM
$-\Delta G_{p}^{S}$	−7.61(2)	−9.00(4)	−8.73(5)	−7.25(4)	−9.28(6)	−9.2(1)
$-\Delta G_{n}^{S}$	−3.81(1)	−1.84(2)	−1.70(1)	−2.25(5)	−2.10(3)	−1.87(3)
$-\Delta G_{o}^{S}$	−2.49(2)	−2.19(2)	−1.38(1)	−2.24(4)	−1.36(2)	−2.21(3)
$-\Delta G_{a}^{S}$	−0.85(1)	−0.34(1)	−0.40(1)	−0.93(1)	−0.44(1)	−0.44(1)
$-k_{\;B}T\;\ln\left(I^{*}S^{*}C^{⊖}\right)$	−28.3(2)	−26.0(3)	−23.9(3)	−24.6(3)	−21.7(3)	−17.5(3)
$\Delta G_{o}^{B}$	6.81	6.81	6.81	6.81	6.81	6.81
$\Delta G_{p}^{B}$	13.1(1)	12.7(2)	11.8(1)	14.1(2)	16.4(2)	15.7(2)
$\Delta G_{n}^{B}$	3.20(3)	3.42(5)	2.05(4)	1.85(3)	1.69(4)	1.89(3)
$\Delta G_{b}^{⊖}$	−20.0(4)	−16.5(6)	−15.5(5)	−14.5(6)	−10.1(6)	−6.8(6)
$\Delta \Delta G_{p}^{B\to S}$	5.5(1)	3.7(2)	3.1(1)	6.9(2)	7.1(2)	6.5(2)
$\Delta \Delta G_{n}^{B\to S}$	−0.62(3)	1.58(5)	0.35(4)	−0.4(1)	−0.4(1)	0.02(4)
$\Delta \Delta G_{o}^{B\to S}$	4.32(1)	4.62(1)	5.43(1)	4.57(1)	5.45(1)	4.6(1)

All units in kcal mol^−1^ with their standard error in parentheses after the last significant digit. Contributions to $\Delta G_{b}^{⊖}$ for all water models and ion concentrations. The last three lines indicate the overall contributions of restricting the peptide $\left(\Delta \Delta G_{p}^{B\to S}\right)$ and the RNA $\left(\Delta \Delta G_{n}^{B\to S}\right)$ conformational freedom and the orientational freedom of the N-peptide upon binding $\left(\Delta \Delta G_{o}^{B\to S}\right)$.

By linear regression it is possible to estimate an overall salt dependence of binding $\left(\partial \Delta G_{b}^{⊖}/\partial \log\left[\text{NaCl}\right]\right)$ yielding 2.9 ± 1.1 kcal mol^−1^ in TIP3P and 4.5 ± 0.6 kcal mol^−1^ in TIP4P-D water.

Experimental data for the salt dependency has been reported for a related system of the bacteriophage λ (24). The two systems have a comparable size with the λ-N-peptide containing 22 residues and a 19 nt RNA versus the present phage 22 N-peptide has 19 residues and the 15 nt RNA. Nevertheless, the experimentally reported salt dependency (5.36 ± 0.19 kcal mol^−1^) (24) agrees reasonably well with the value obtained with TIP4P-D water for the N-peptide/RNA system.

Note, binding affinity experiments have also been conducted independently by Van Gilst et al. for the homologous N-peptide boxB-RNA complex of λ-phage by steady-state intrinsic fluorescence measurements. These authors obtained an experimental binding affinity $\Delta G_{b}^{\exp}$ of −12.2 ± 0.2 kcal mol^−1^ for an approximate ion concentration of 120 mM. Our regression analysis predicts that an ion concentration of 127 ± 4 mM would correspond to their $\Delta G_{b}^{\exp}$ of −12.2 ± 0.2 kcal mol^−1^, which agrees well with their ion setup (68).

**Figure 4:**
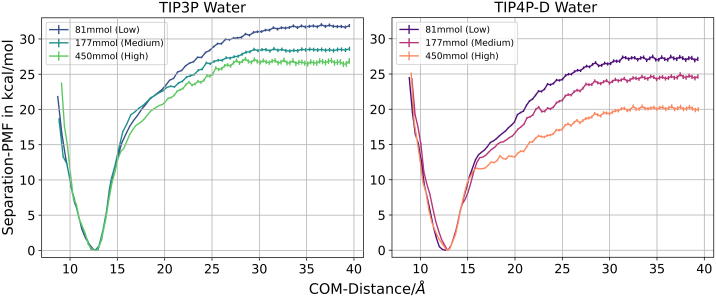
Comparison of the potential of mean force curves with standard errors obtained from the WR method. Standard errors of the mean are depicted as bars.

However, the results with the TIP3P water model clearly overestimate the binding free energy, which is due to a larger PMF by about 4–6 kcal mol^−1^ (see Fig. 4) but also due to a smaller free energy penalty of forming the bound helical structure of the N-peptide by 1–3 kcal mol^−1^ (compared with TIP4P-D) (see Table 1).

Overall, the free energy for the N-peptide to adopt the conformational ensemble representing the bound state is unfavorable and opposes binding at all salt concentrations by 3–5 kcal mol^−1^ for TIP3P water and by 6–7 kcal mol^−1^ for TIP4P-D. This difference likely arises from the enhanced solute-solvent attraction of the TIP4P-D water model and its propensity for disordered peptide conformations (29). At the same time, TIP3P water has been reported to overstabilize helical protein conformations, which explains the discrepancy concerning the peptide folding penalty (69).

Interestingly, the restriction of the conformational freedom of the RNA partner contributes little to overall binding (Table 1). Hence, its unbound structure remains close to the conformation in the bound complex. The good agreement of the calculated absolute binding free energies with experiment in the case of the TIP4P-D water model gives confidence in the presented force field approach. Also, the simulations give some insight into the free energy contributions for the coupled folding and binding process (e.g., restriction of conformational and orientational freedom). However, possible intermediate states, preorientation or partial folding of the peptide before contact formation cannot be deduced due to the use of orientational and conformational restraints during the absolute binding free energy simulations.

### Induced dissociation/association without geometric and conformational restraints

To study the process of dissociation/association of the N-peptide boxB complex, HREUS simulations along the axis between the centers-of-mass of the RNA’s and the peptide’s heavy atoms were conducted. No further conformational or orientational restraints were included. This enables the analysis of the conformational states visited during the unrestrained process. Since extensive sampling of relevant conformational states is necessary at every US sampling step, considerably longer simulation times are required than for the WR method (see previous section, and methods).

**Figure 5:**
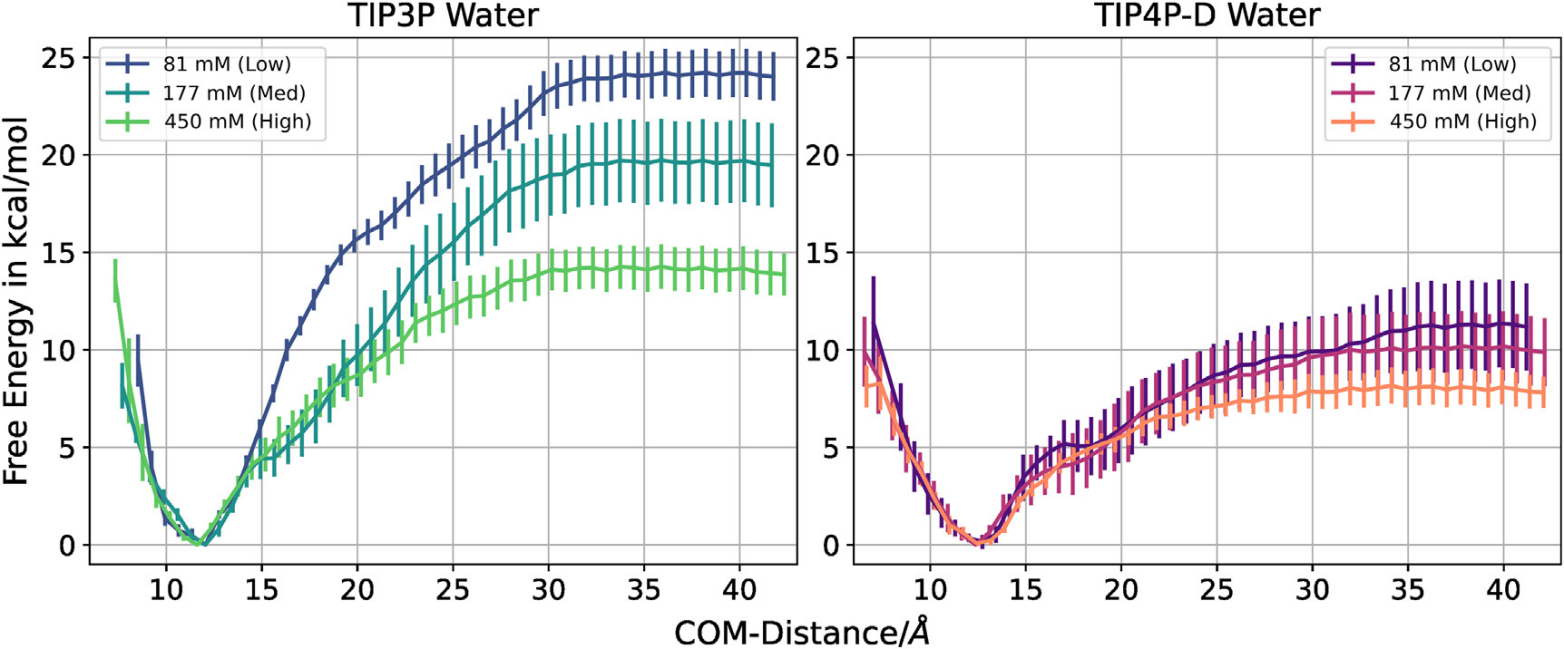
Calculated free energy change along the N-peptide boxB separation center-of-mass distance coordinate using the TIP3P or TIP4P-D water models and three salt concentrations. No additional restraints on conformation or partner orientation are included. Standard errors of the mean are depicted as bars.

In this case, it is also possible to calculate absolute binding free energies which differ from those obtained with the WR method but follow a similar qualitative trend (Fig. 5; Table 2).

**Table 2:** Summary of $\Delta G_{b}$ values obtained from conventional US simulations along the center-of-mass distance coordinate

Contribution	TIP3P			TIP4P-D		
	81 mM	177 mM	450 mM	81 mM	177 mM	450 mM
$-k_{\;B}T\;\ln\left(I^{*}C^{⊖}\right)$	−20.0 ± 1.4	−15.4 ± 2.2	−9.9 ± 1.3	−7.2 ± 5.2	−6.0 ± 2.0	−4.0 ± 1.1

All units in kcal mol^−1^ with their standard error in parentheses after the last significant digit. The separation contribution $I^{*}$ is the only contributor to $\Delta G_{b}$ and is listed for clarity.

The calculated binding free energy in case of TIP3P is again larger in magnitude compared with the TIP4P-D results and it decreases with increasing salt concentration. Interestingly, at the lowest salt concentration it agrees quite well with the results obtained with the WR method and overestimates the binding affinity compared with experiment. Nevertheless, our TIP3P salt dependence of 5.89 kcal mol^−1^ is in quite close agreement to experiment (24) with 5.36 kcal mol^−1^ per log unit change in salt concentration. In the case of using the TIP4P-D model the calculated binding free energy is lower than the experimental reference and also slightly smaller than for the WR method. It may indicate still incomplete convergence of the calculations. However, our main goal in this section is not to achieve complete convergence of thermodynamic quantities but rather to characterize the process of complex formation/dissociation.

Both water models yield at all salt concentrations a deep minimum at ∼11 Å, indicating a similar geometry of the bound complex. The association/dissociation phase starts at ∼15 Å and yields a steadily decreasing slope of the free energy change. The onset of the dissociated regime, characterized by a nearly constant free energy starts at slightly smaller distances with increasing salt concentration and settles at lower free energy levels. An approximate threshold of 33 Å can be defined. No free energy barrier is involved, indicating a barrierless approach of the peptide toward the RNA binding region. It is interesting to note that it is possible to fit the PMF regime beyond a distance of 20 Å quite accurately by a simple Debye-Hueckel model (see Fig. S3). Hence, it indicates that beyond this distance the interaction is mainly driven by electrostatics and can be described by a simple Debye-Hueckel screening approach.

### Axial distribution of N-peptide at different distances from boxB RNA

**Figure 6:**
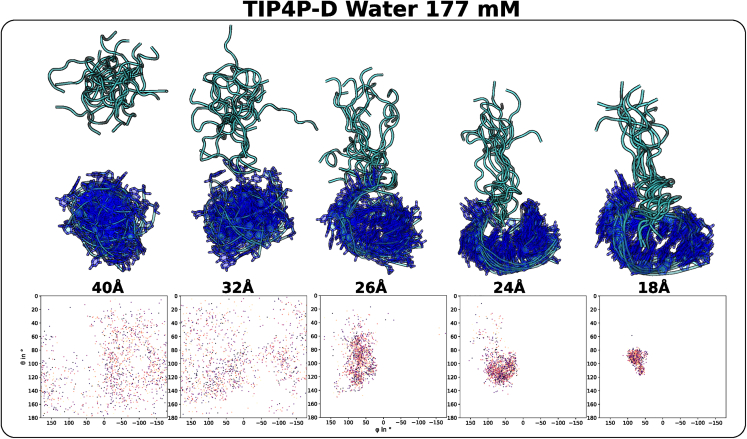
Spherical distribution (in terms of the polar and azimuthal angles θ and ϕ) of the N-peptide relative to the boxB RNA at different center-of-mass distances. The panels above each plot illustrate the distribution of peptide (*cyan*) and RNA (*blue*) partners by best superposition of representative snapshots. The distribution and relative orientation becomes increasingly nonrandom with decreasing center-of-mass distances (see also Figs. S4–S7).

To characterize any possible positional or orientational preferences of the peptide before or upon approaching the boxB RNA, we first investigate the distribution of the peptide center-of-mass position around the RNA target at different separation distances. For a given separation window, the distribution is characterized by the spherical angles θ and ϕ of the peptide-RNA center-of-mass vector. Here, θ and ϕ correspond to the system’s polar and azimuthal angle instead of to the restraint angles mentioned in Fig. 2. For separation distances above 35 Å, a wide and relatively even distribution in the spherical angles was obtained (see Figs. 6, S4, and S5). However, for smaller distances (below 32 Å) the picture changes and partial accumulation of the N-peptide placement relative the boxB hairpin is observed. At a center-of-mass distance of 26 Å (and at smaller distances) an attractive encounter region is reached including contacts to the nucleotides that form the RNA hairpin and the entrance of the native binding region that corresponds to the RNA major groove below the hairpin nucleotides (Fig. 6).

It is also interesting to note that the attractive regime observed in the calculated PMF starting at distances smaller than 26 Å reaches into the regime where the N-peptide is still outside the encounter binding site (see Fig. 5) and also can be split into two separate encounter regions (Figs. S4 and S5). Similar conclusions can be drawn from the patterns observed at other salt concentrations (see Figs. S6 and S7).

### Analysis of the orientational preference of the N-peptide upon complex formation

**Figure 7:**
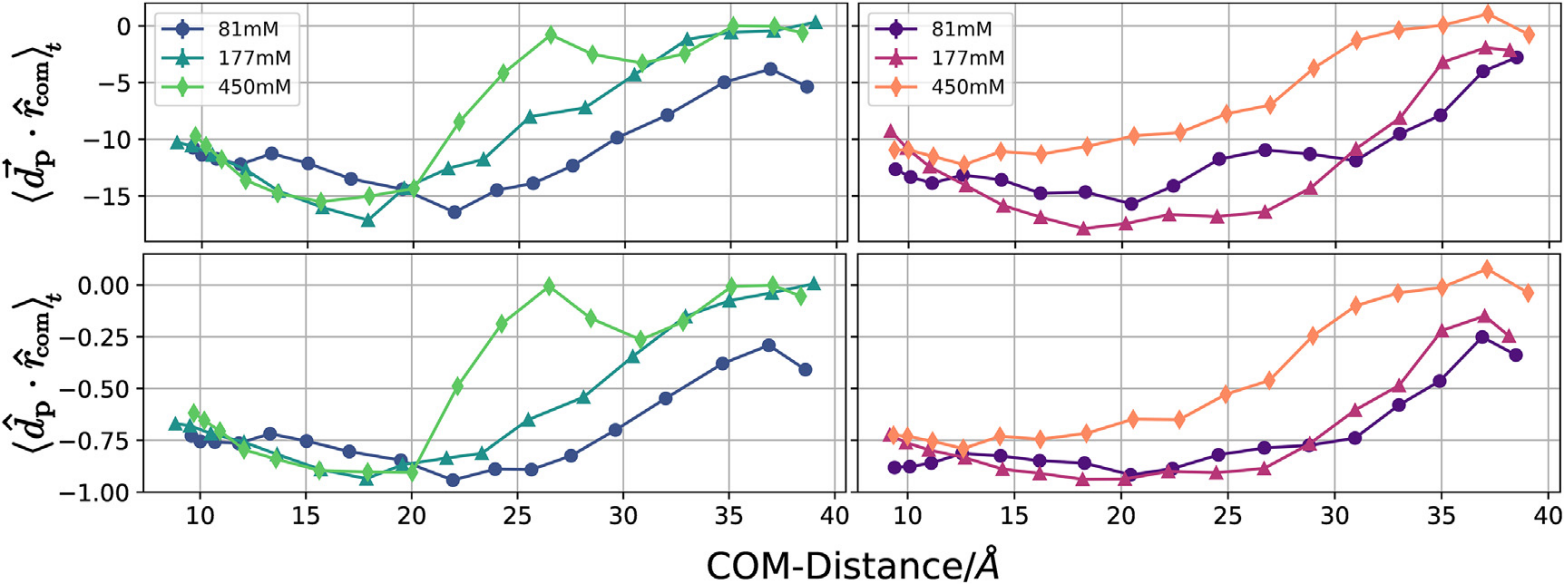
Rolling time-average of the dot products between the peptide’s dipole moment (calculated relative to the center-of-mass of the peptide) and the normalized center-of-mass vector (*upper panels*) and between the normalized dipole moment vector and the normalized center-of-mass vector. A stepsize of 5000 frames and a window size of 20000 frames was used. In the latter case only the direction and not the magnitude of the dipole vector is considered. Standard errors of the mean are depicted as bars.

One of the first questions that arises is if the peptide and RNA are still oriented toward each other even for very high distances. For that purpose, the time-averaged dot product between the peptide’s dipole moment and the center-of-mass vector between the RNA and peptide was calculated (see Fig. 7). The dipole moment of a charged system depends on the choice of the reference origin. Here, it is calculated with respect to the center-of-mass of the peptide. This is reasonable since the peptide center-of-mass is used to steer the complex apart or together. A negative value for the projection on the RNA-peptide center-of-mass vector indicates that the positive charge of the peptide points toward the negative RNA. For distances below 30 Å, a clear orientational preference is observed that gradually vanishes at larger distances and more rapidly at the highest ion concentration. Due to high fluctuations of the relative peptide orientations and limited sampling, but also due to the fact that not only the electric field but also fluctuating intermediate contacts of the peptide and RNA influence the mean relative orientation a, clear sequential order with respect to the ion concentration is not observed. However, at the low salt conditions, a clear overall orientational preference of the peptide charge distribution is observed. Distinctly so in the regime where the N-peptide is dissociated from the RNA (r> 30 Å center-of-mass distance).

**Figure 8:**
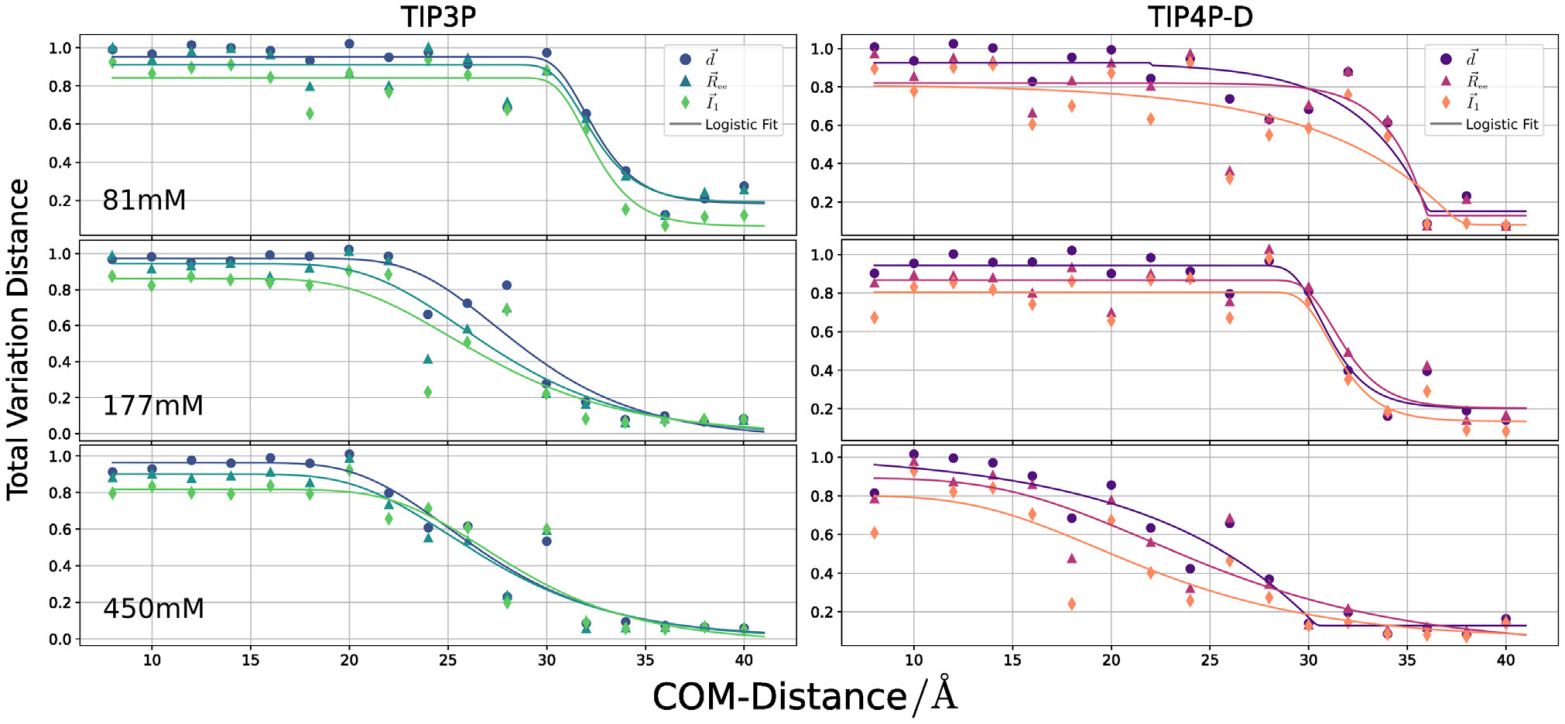
Total variation distance $D_{\;\text{TV}}$ between bulk orientations and orientations adopted during the US simulations. Low values for $D_{\;\text{TV}}$ correspond to bulk-like behavior and indicate the absence of ordered configurational ensembles. Boundaries, $\Xi^{D_{\text{TV}}}$, are extracted as the middle of three columns with consistently increasing $D_{\;\text{TV}}$ for decreasing $r_{i}$. For example, for TIP3P water with low salt concentration, this would be 34 Å. A generalized logistic curve is fitted to each data set as a line (70).

The process of N-peptide structure formation and orientational preference can also be captured by the total variation distance $D_{\;\text{TV}}$ (see Eq. (3)) of structural parameters. It can vary between 0 (no preference relative to bulk) and 2 (maximal preference). We use three distinct structural vectors, $\Omega^{I_{1}}$, $\Omega^{d}$, and $\Omega^{\text{ee}}$, that correspond to the principal axis ${\vec{I}}_{1}$, the dipol moment $\vec{d}$ and the end-to-end-vector ${\vec{R}}_{\;\text{ee}}$, respectively (Fig. 8). Due to the narrow distance regime for the transition and coupled conformational as well as orientational changes, especially for the TIP4P-D model the fitted plots in Fig. 8 reflect trends in the order parameters.

At all salt concentrations and for both water models, little orientational preference of the peptide is observed above separation distances of 35–40 Å (but still a slight preference of the dipole moment to point toward RNA, see previous paragraph). At the lowest salt concentration, a rapid transition to a nonbulk distribution is observed at a distance interval of 35 down to 28 Å (Fig. 8). At higher salt concentrations this transition regime becomes broader and the onset starts at 32 Å and goes down to 20–25 Å at 177 mM and down to 15–20 Å at 450 mM. The same trends are observed for both water models. Alternatively, one can also look at the orientational RMSD (the RMSD of the peptide with respect to the structure in the complex without rotational alignment after best superposition of all trajectory frames on the RNA in the reference complex, see Fig. S8). It also indicates onset of peptide orientation relative to RNA at distances of 32 Å. For center-of-mass distances below 15 Å, the structure and orientation of the N-peptide further approaches the experimental reference.

Conclusively, from these plots a prefolding distance boundary $\Xi^{D_{\text{TV}}}$ is derived. $\Xi^{D_{\text{TV}}}$, which signifies the distance of complex orientation onset, when viewed as an association or the transition into bulk-like behavior with respect to a dissociation point of view. The transition distances are listed in Table 3 together with boundaries extracted from other measurements.

**Table 3:** The boundaries of early binding and folding of the N-peptide and boxB RNA as a measure of center-of-mass distance

System	$\Xi_{\vee }^{\delta r}$	$\Xi_{\vee }^{f}$	$\Xi_{\wedge }^{\delta r}$	$\Xi^{D_{\;\text{TV}}}$	$\Xi_{\wedge }^{f}$
TIP3P low	26	28	32	34	34
TIP3P medium	24	28	30	30	34
TIP3P high	24	24	28	28	36
TIP4P-D low	22	28	34	34	38
TIP4P-D medium	26	26	32	32	38
TIP4P-D high	22	22	32	28	40

All measures in Å are approximate with an estimated uncertainty of ±1. Upper boundaries with respect to a measurement are depicted with ˄, lower boundaries with ˅. The boundaries are sorted by magnitudes.

### Formation of initial contacts upon complex formation

**Figure 9:**
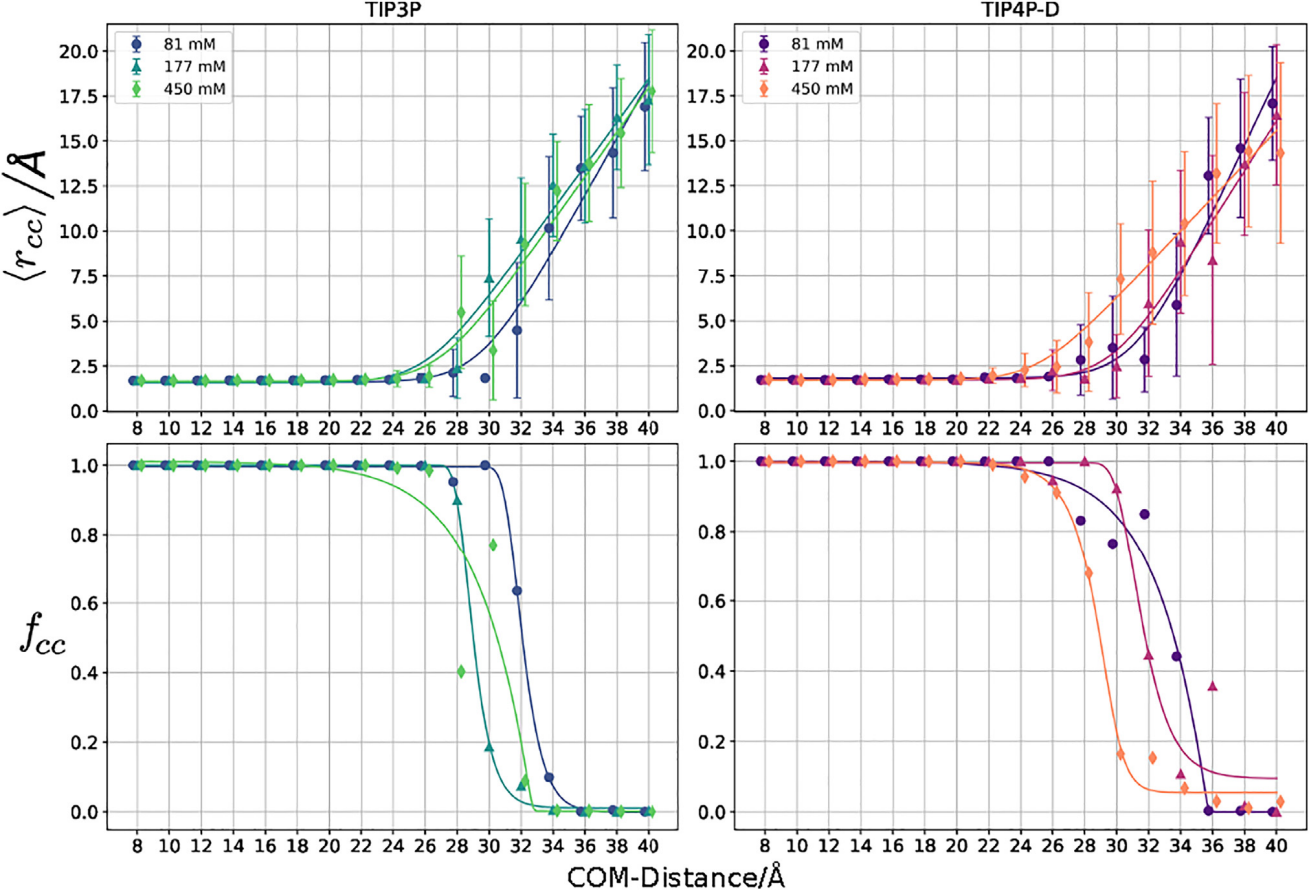
Upper panels: average closest contact distance $\left\langle r_{\text{cc}}\right\rangle$ with error bars corresponding to $\delta \left\langle r_{\text{cc}}\right\rangle$ versus RNA-peptide center-of-mass distance. The lines depict a fitted function of the form f(x)=a+blog(cexp(x−d)+e). Lower panels indicate the frequency of a contact closer than 4 Å, $f_{\text{cc}}$. Here the line depicts a fitted, generalized logistic curve (70).

After identification of the separation distances that initiate axial and orientational preferences of the N-peptide relative to the boxB RNA we investigated the frequency of peptide-RNA contacts, $f_{\text{cc}}$, and the average closest contact distance, $\left\langle r_{\text{cc}}\right\rangle$. The $f_{\text{cc}}$ depicts the frequency with which contacts smaller than 4 Å form along the reaction coordinate (see Fig. 9).

In the regime below 26 Å, $\left\langle r_{\text{cc}}\right\rangle$ remains constant for both water models until it expectedly increases linearly with the center-of-mass distance. Also the fluctuations $\delta \left\langle r_{\text{cc}}\right\rangle$ remain very low for low center-of-mass distances and then increase up to ∼10 Å. At high salt concentration the plotted line is slightly shifted to smaller center-of-mass distances. As expected, the $f_{\text{cc}}$ measurements exhibit two constant regions. For low distance values, the $f_{\text{cc}}$ is equal to one, since every frame will feature close contacts, and for large center-of-mass distance values $f_{\text{cc}}$ is zero (Fig. 9). In general, the higher salt concentrations develop diminishing $f_{\text{cc}}$ values at smaller distances. The transition between no sampled close contacts and $f_{\text{cc}}$ close to one occurs within a narrow distance regime of a few Å. The values for the upper and lower boundaries derived from $\delta \left\langle r_{\text{cc}}\right\rangle$, $f_{\text{cc}}$, and $D_{\;\text{TV}}$ are summarized in Table 3.

Some of these boundaries might be redundant, e.g., $\Xi^{D_{\;\text{TV}}}$ coincides very well with $\Xi_{ˆ}^{\delta r}$. This finding is expected, since both boundaries relate to the orientational freedom of the N-peptide relative to the RNA.

### Structural deviation of the N-peptide and RNA from the bound conformation

**Figure 10:**
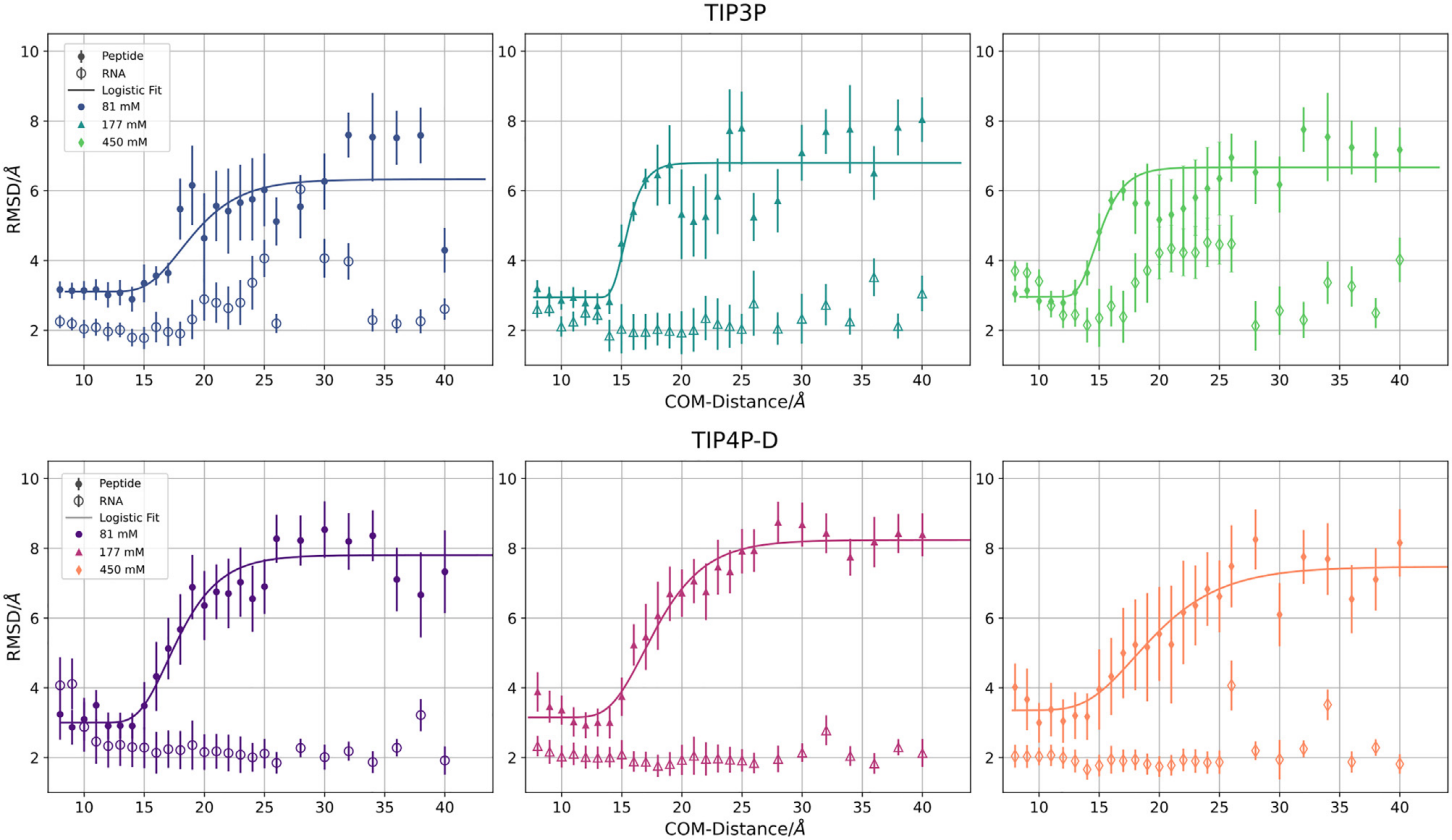
Average deviation of the N-peptide and boxB RNA conformation from the native bound structures along the HREUS center-of-mass distance coordinate. The lines represent a generalized logistic function fit (70). The average RMSD was calculated for all nonhydrogen atoms with respect to the individual peptide and RNA conformation in the experimental complex structure. Standard errors of the mean are depicted as bars.

Besides the onset of axial and orientational preferences, the conformational adaptation during the binding process is of interest. Interestingly the N-peptide adopted on average conformations with significant deviation from the bound peptide structure already at separation distances of above 15 Å and showed a plateau at a distance of 20–25 Å, with an RMSD level of 7 Å in case of the TIP3P water and ∼8 Å for the TIP4P-D water model (see Fig. 10).

Similar trends concerning the peptide’s conformational transition to its bound state were found for both water models (see Fig. 10). This indicates that a fully folded peptide structure is adopted only close to the final stage of the binding process (below center-of-mass distance of 20 Å). The RNA structure remains relatively close to the bound structure (RMSD smaller than 2.5 Å), except in case of the TIP3P water model where transient conformational changes in the hairpin loop region were observed at intermediate center-of-mass distance regions (reflected in slightly enhanced RMSD of the RNA, see Fig. 10).

The onset of hydrogen (H)-bonds and native contacts follows a similar pattern as observed for changes in RMSD and other parameters discussed above. In the case of the simulations with the TIP3P water model, the first H-bonds between peptide and RNA are formed already at a center-of-mass distance of 30 Å and a steady increase for smaller distances to reach a maximum at the distance that corresponds to the bound complex (see Fig. S9). The onset of H-bond formation starts at approximately the same distances for each salt concentration. In contrast, native contacts start to form at smaller distances of 25 Å; hence, the initial H-bonds are nonnative contacts. However, in case of the TIP4P-D simulations we observe already onset of native contacts at a separation distance of 30–35 Å followed by the formation of H-bonds at smaller distances of 25 Å. Note, the definition of a native contact is not binary unlike an H-bond. Hence, some of the early bumps in $Q_{\text{nat}}$ could correspond to the peptide’s C-terminus traversing in the vicinity of the RNA loop bases.

Overall the onset of the observed native contact and intermolecular H-bond formation correlates well with the start of the N-peptide conformational transition toward the bound structure at a distance of approximately 25 Å (see Fig. 10). Below this distance both the deviation from the native peptide structure and the formation of H-bonds and native contacts correlate. This indicates that the contacts support and guide the peptide conformation toward the native bound structure.

**Figure 11:**
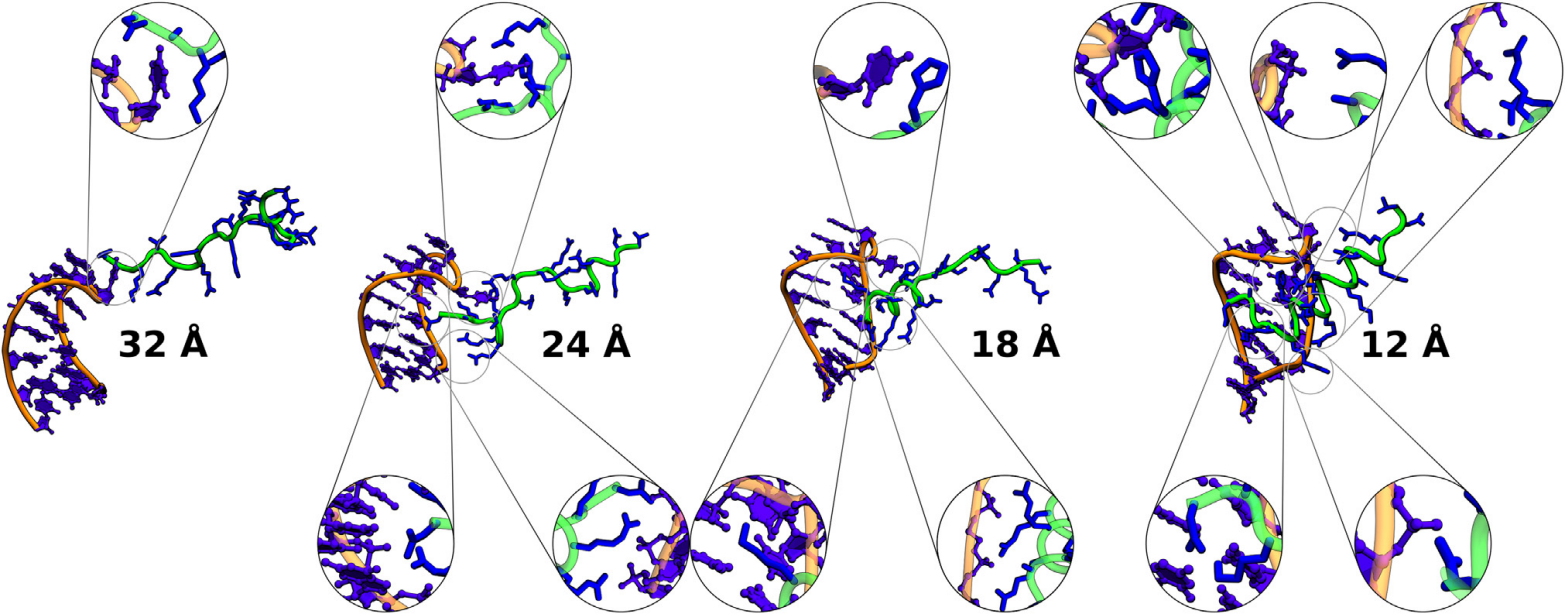
Representative binding process rendered for the TIP4P-D water model with 81 mM salt concentration. The peptide approaches from the top of the hairpin loop and folds into the major groove over time. The RNA and peptide are shown in their usual color schemes. The RNA atoms are represented via balls and sticks, while the peptide side chains are shown as licorice in the detail plots.

It is also interesting to look at the initial contacts formed between N-peptide and RNA (or the last contacts that are possible upon dissociation). The central C8 loop base contacts the N-peptide at the largest distance where contacts are still possible for all ion concentrations. It forms an H-bond with ASN1 and also contacts the LYS3, which contacts the negatively charged phosphate backbone. The ASN1 also contacts the base and sugar of the A7 loop nucleotide (structure 32 Å illustrated in Fig. 11 for the TIP4P-D model). At this stage, all other residues are solvent exposed. The next phase in the binding is marked by ARG5 and ARG6 forming salt bridge contacts with the phosphate backbone of nucleotides C2, G3, and C4. Furthermore, the ASN1 moved into the major groove contacting the base oxygen of C3 and the amine group of C4. Its N-terminus also interacts with the base oxygens of G6 and U5 and the phosphate group of A9. The A7 loop base is still quite relevant and contacts LYS3, ARG9, HIS7, and several backbone atoms (structure at 24 Å Fig. 11). With further decreasing center-of-mass distance ARG9 joins ARG5 and ARG6 in contacting the phosphates of C2, G3, C4, and U5. LYS3 also relocates closer to the major groove, and forms salt bridges with the phosphate groups of A9 and A10. Another prominent triple contact is formed between C8 and HIS7 and THR4. Also the sugar of C8 seems to stabilize the alkyl chain of LYS3 (see 18 Å structure of Fig. 11). At a center-of-mass distance of ∼12 Å the binding process is basically complete as is the peptide helix formation. The C8 loop base contacts the HIS7 and the ARG11. The HIS7 furthermore contacts the C8 sugar and the A9 phosphate group. The ARG17 contacts the C8 phosphate group. ARG5, ARG6, and ARG9 still contact the right-hand-side phosphate backbone (G3, C4, and U5). In the major groove, the ASN1 N-terminus contacts the A11 and G12 bases and the LYS3 forms contacts with the C13, G3, and C4 bases, and the ASN1 side chain (see 12 Å structure of Fig. 11).

Although, there are many subtle differences in the structure, the common pattern remains that the N-peptide approaches the boxB RNA from the upper right side (5′-GAC-3′) before sliding into the bound minimum with strong backbone binding via ARG5, ARG6, and ARG9 among other binding motifs.

## Discussion

The binding of peptides rich in basic residues to RNA targets is driven by the strong electrostatic attraction and frequently involves the transition of a largely disordered peptide to a defined, often α-helical, bound conformation. One useful model system for studying such binding process is the P22 N-peptide-boxB RNA complex. The aim of our MD simulation/US study was, firstly, to characterize the thermodynamic driving forces and contributions that favor or disfavor N-peptide-boxB binding. Secondly, we analyzed the axial distribution and onset of orientational as well as conformational preferences of the binding partners along a distance separation coordinate.

The calculation of absolute binding free energies including the salt dependence resulted in good agreement with experiment for the simulations involving the TIP4P-D water model, whereas the TIP3P model overestimated the binding free energy. The TIP4P-D model was specifically designed to enhance the interaction of solutes with the solvent to reduce the (over)stabilization of proteins by other water models (29,71). Hence, the smaller but more realistic, calculated binding free energy with the TIP4P-D model is due to the reduced PMF along the center-of-mass distance (TIP4P-D stabilizes the separated solvated state) and due to a stronger stabilization of the unfolded peptide compared with the TIP3P model observed in our study. Our calculations indicate that the transition of the disordered N-peptide toward the bound structure strongly opposes binding by 5–6 kcal mol^−1^ depending slightly on the salt concentration. Also, the calculated salt dependence of peptide-RNA binding agrees quite well with experiment in case of the TIP4P-D model. In addition, free energy contributions to restrict axial and orientational freedom of the peptide also oppose binding significantly by 4–5 kcal mol^−1^ for both water models. Interestingly, the restriction of the conformational freedom of the RNA did not contribute significantly to binding. However, the opposing contributions are dwarfed by the strongly attractive PMF along the separation coordinate.

The analysis of US simulations without any additional restraints indicated detectable axial preferences at distances that involve first peptide-RNA contact formation. However, we observed a significant orientational preference of the positive peptide charges to point toward the RNA already at distances that do not allow contacts. The accumulation of axial peptide placement correlates with the formation of contacts between N-peptide and RNA that involves mostly nucleotides of the hairpin loop. The broad initial regime becomes narrower relative to the final placement at decreasing distance to the binding site. Further analysis of the orientational preference of the N-peptide relative to RNA by several measures suggests that a strong preorientation toward the orientation in the native complex starts simultaneously with the first contact formation at ∼35 Å. Note that possible nonequilibrium effects of the fast peptide RNA approach that do not allow for orientational and conformational equilibration during the process are not considered in the US simulations. From the plots, intermediate structures could be extracted, which showed that the complex association is mostly steered by arginine backbone binding. Especially, ARG5 and ARG6. ARG9 and ARG10 seem to be important, as well as the C8 of boxB RNA. The number of contacts increases up to a distance of 20 Å, such that the peptide adopts an orientation close to the one observed in the native complex. The transitions occur smoother and over a longer distance regime and also start at smaller distances for the higher salt concentrations compared with less salt. However, this correlates also with an earlier onset of contact formation at the lower salt concentration and is therefore not a direct electrostatic effect. Significant formation of native contacts also starts at ∼20 Å and correlates with the decrease of the RMSD of the peptide with respect to the bound structure that also begins at a distance of ∼20 Å. Hence, the full structural adaption toward the native α-helical N-peptide conformation occurs mostly at a final stage with several H-bonds and native contacts already formed.

## Acknowledgments

This work was performed within the framework of the SFB 1035 (10.13039/501100001659German Research Foundation, Sonderforschungsbereich 1035, Projektnummer 201302640, project B02). The authors thank Friederike Schubert for helpful discussions. We acknowledge HPC resources provided by the Erlangen National High Performance Computing Center (NHR@FAU) of the 10.13039/501100001652Friedrich-Alexander-Universität Erlangen-Nürnberg (FAU) under the 10.13039/501100008084NHR project B118BB. 10.13039/501100008084NHR funding is provided by Federal and Bavarian State authorities. NHR@FAU hardware is partially funded by the 10.13039/501100001659German Research Foundation (10.13039/501100001659DFG) – 440719683.

## Author contributions

L.V. performed research, analyzed data, contributed analytic tools, and wrote the article. M.Z. designed and supervised the research and wrote the article.

## Declaration of interests

The authors declare that they have no competing financial interests or personal relationships that could have appeared to influence the work reported in this paper.

## Supporting material

Document S1. Figures S1–S9

Document S2. Article plus supporting material
