# Supplementary material for: Advanced sampling simulations of coupled folding and binding of phage P22 N-peptide to boxB RNA
Source: Biophys J. 2024 Aug 28;123(19):3463–77. doi: 10.1016/j.bpj.2024.08.022 (PMC11480772; doi:10.1016/j.bpj.2024.08.022)
Supplement: Document S1. Figures S1–S9 [file mmc1.pdf]

Biophysical Journal, Volume *123*

Supplemental information

Advanced sampling simulations of coupled folding and binding of phage P22 N-peptide to boxB RNA

Luis Vollmers and Martin Zacharias

Supporting Information: Advanced Sampling Simulations of Coupled Folding and Binding of Phage P22 N-Peptide to BoxB RNA

Luis Vollmers[†,‡] and Martin Zacharias[*,†,¶]

†*Physics Department and Center of Protein Assemblies, Technical University Munich*

‡*85748 Garching, Germany*

E-mail: zacharias@tum.de

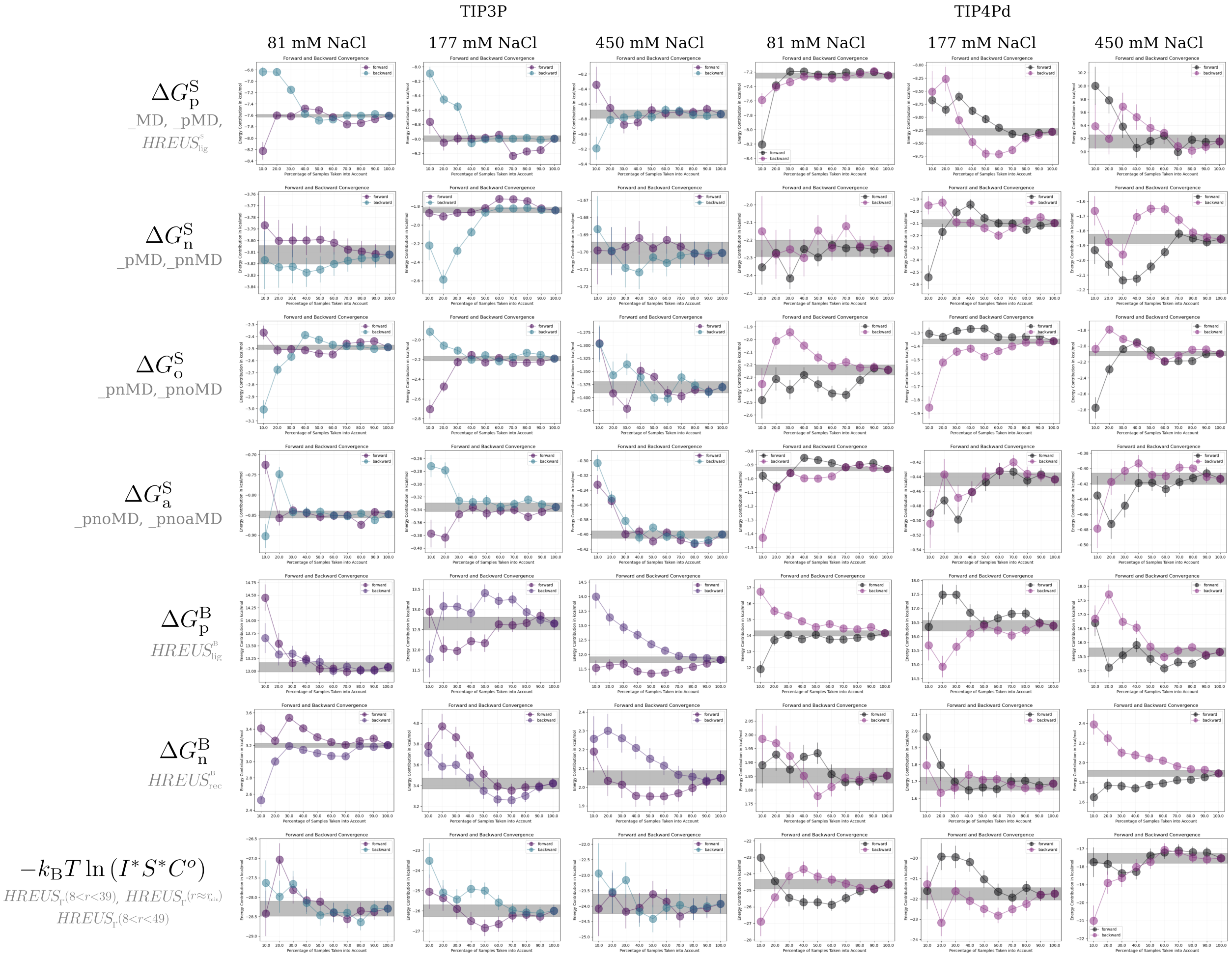

Figure S1: Forward-backward-convergence for all free energy contributions as defined by Klimovich et al.[1] After decorrelation, each energetic contribution is calculated with only 10% of the simulation data, then 20%, then 30% and so on. First, this process starts out from the beginning of the time series (forward) and thereafter, the calculation starts with the last 10% of the time series (backward). A fast collapse of the resulting data points within the standard deviation of the final value (grey band) indicates a good convergence of the free energy contribution.

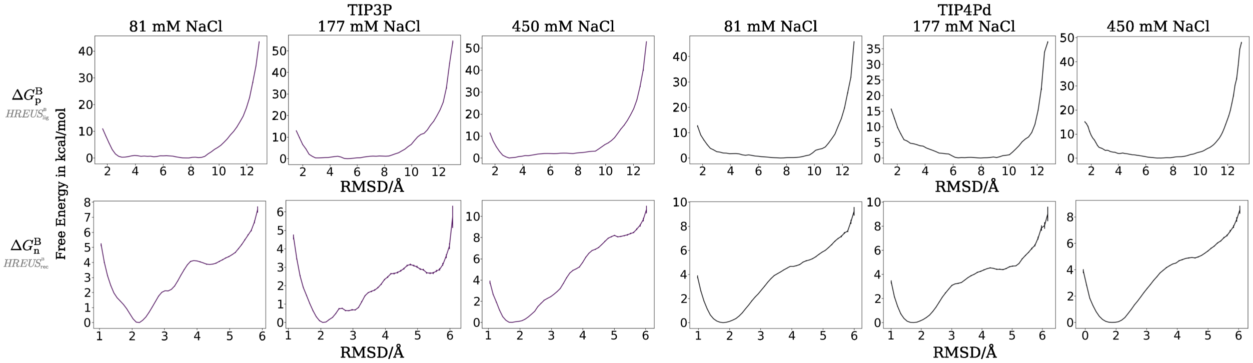

Figure S2: Calculated potentials of mean force along the RMSD (only non-hydrogen atoms) with respect to the bound conformation of the N-peptide (upper row) and the boxB RNA (lower row of plots) in the bulk. The HREUS calculations along the RMSD coordinate of the RNA were performed in steps of 0.3 Å in the range of 1 to 5.5 Å with a force constant for the quadratic potential of $10\,\mathrm{kcal\,mol^{-1}\,Å^{-2}}$. Replica-exchange attempts were performed every 1 ps with an acceptance ratio of > 0.1. For the peptide asymmetric steps were chosen in a range of 1 to 15 Å.

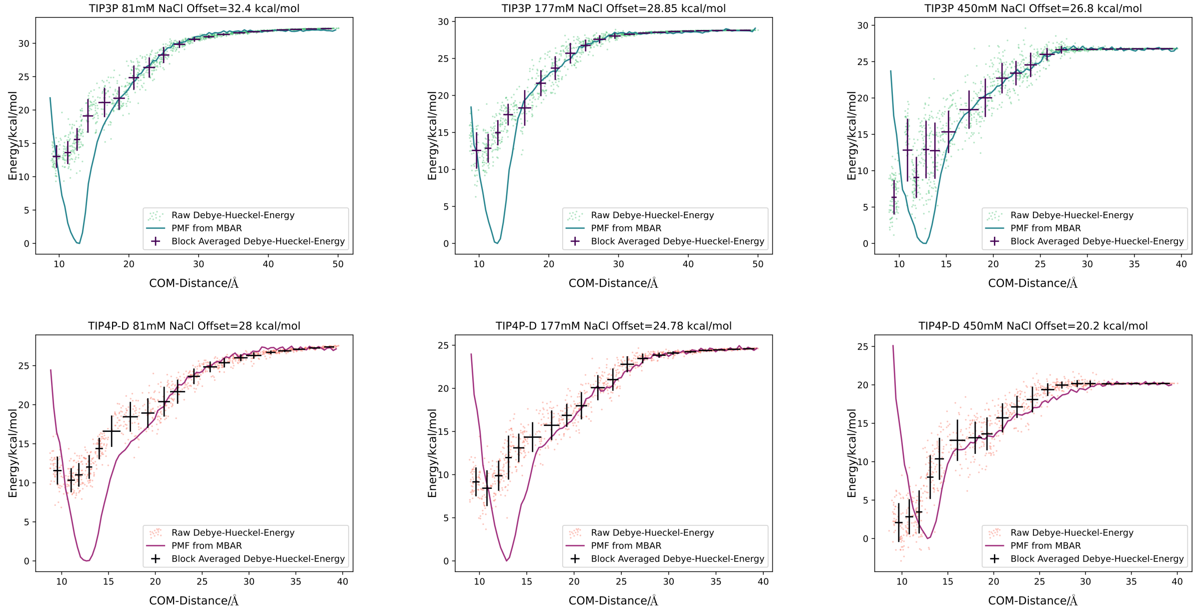

Figure S3: Calculated electrostatic interaction energy according to the Debye-Hueckel equation for each salt concentration (and water model). The raw Debye-Hueckel energies were calculated from snapshots of the corresponding simulation trajectories. The block averages were then calculated in a way that 20 data points emerge with standard deviations in x- and y-direction. The PMF curves are the same as shown in the main document. Approximately, for center-of-mass-distances above 20 Å the separation PMF can be represented by the Debye-Hueckel model quite accurately.

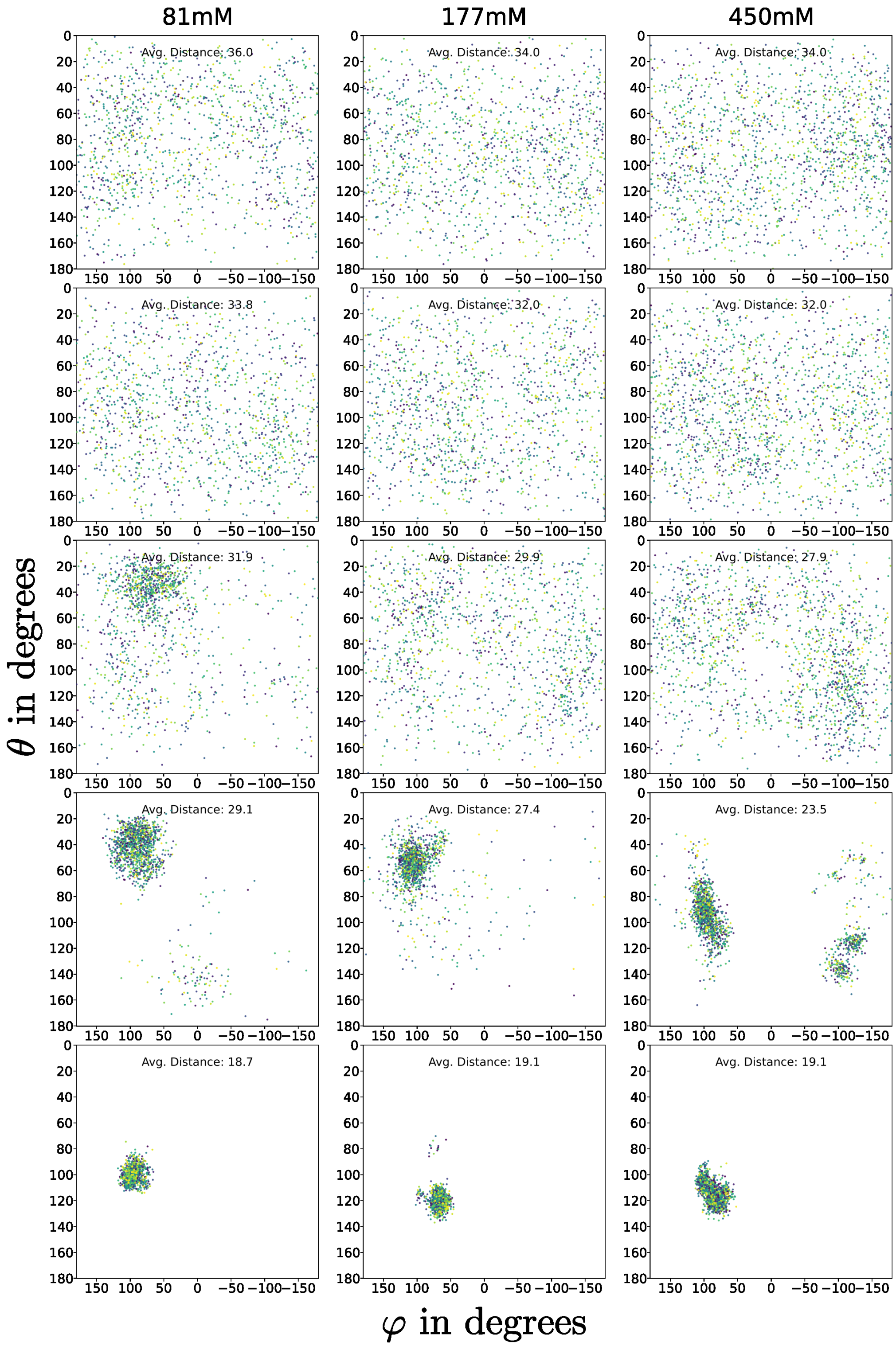

Figure S4: Distribution of the system's polar and azimuthal angles θ and φ indicating the axial N-peptide center-of-mass placement relative to the RNA for the umbrella windows illustrated in S6. Each point is a trajectory frame and the color reflects the peptide-RNA center-of-mass-distance within the respective window. For large distances (upper panels) the distribution is nearly random but with decreasing center-of-mass-distances axial placements increasingly mimic near native binding placement.

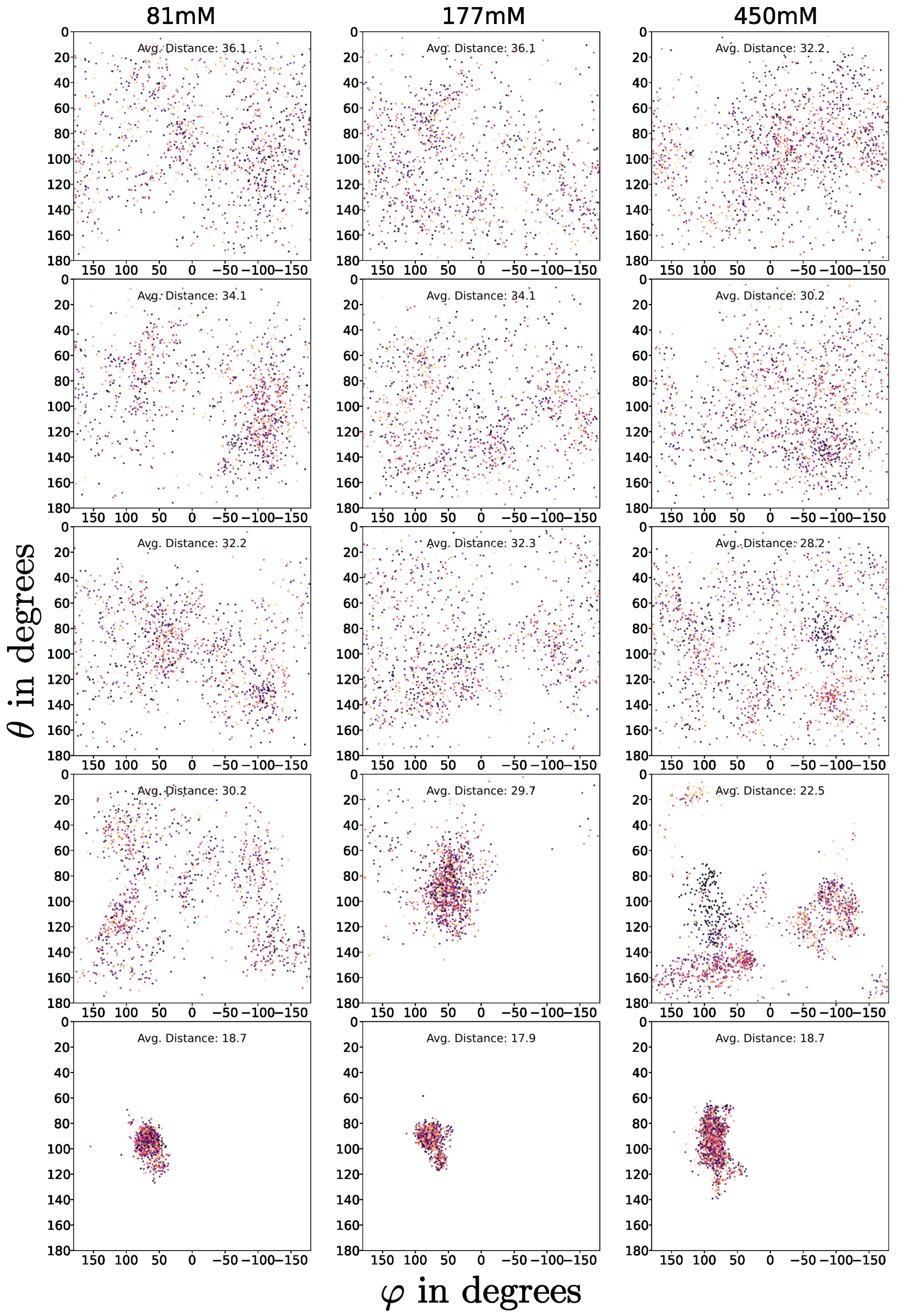

Figure S5: Distribution of the system's polar and azimuthal angles θ and φ for the umbrella windows visualised in S7. Each point is a trajectory frame and the color reflects the peptide-RNA center-of-mass-distance within the respective window. For large distances (upper panels) the distribution is nearly random but with decreasing center-of-mass-distances axial placements increasingly mimic near native binding placement.

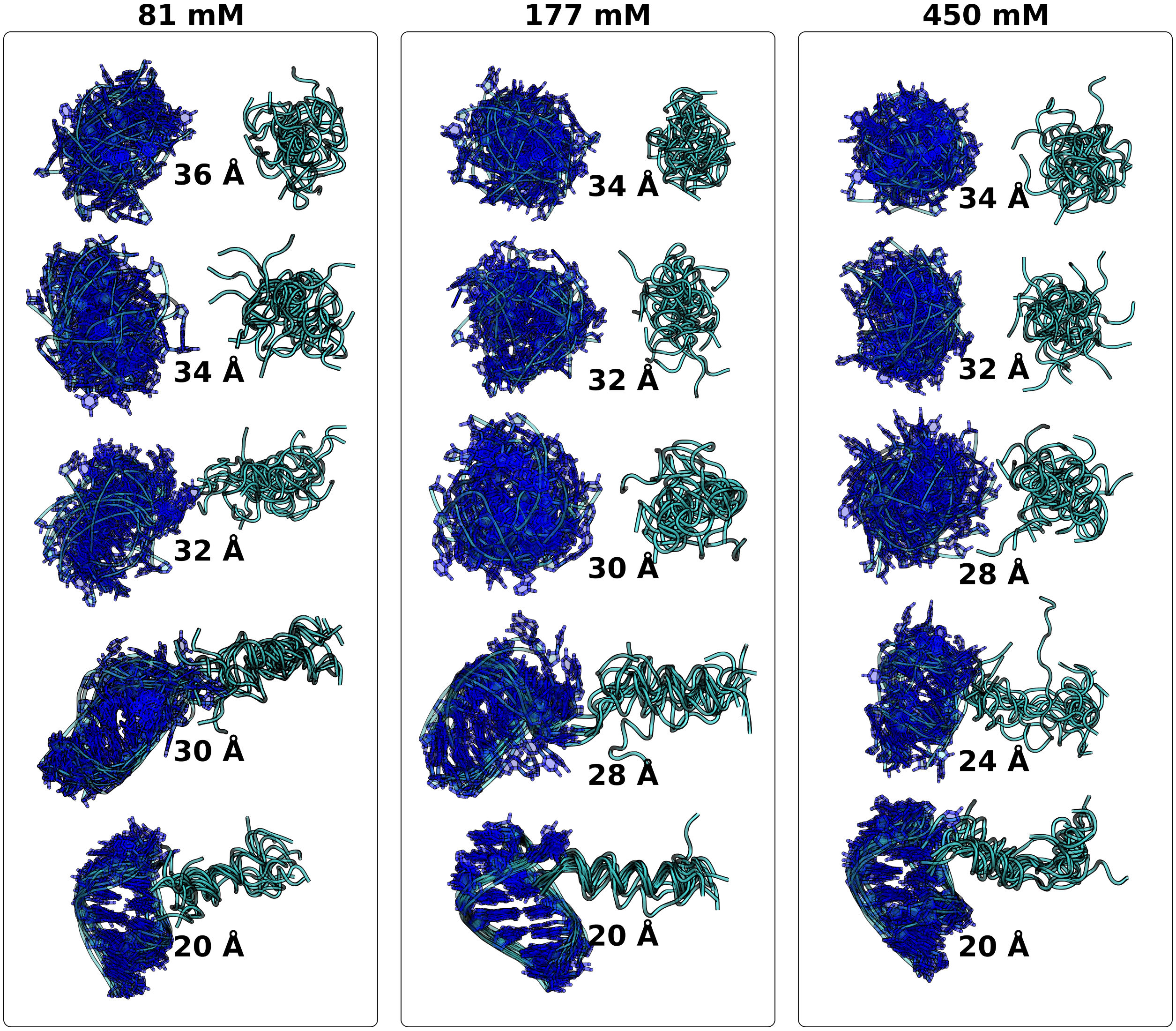

Figure S6: Visualisation of peptide orientations for different center-of-mass-distances (indicated in bold font) for the TIP3P water model. The peptide and RNA coordinates were taken as snapshots from the corresponding US window and were rotationally and translationally aligned with respect to the RNA-peptide center-of-mass-vector to illustrate the peptide and RNA orientation. For higher center-of-mass-distances the orientations of the peptide (cyan cartoon) and RNA (blue cartoon) are relatively random while they become more and more defined with decreasing distances and the formation of contacts to the RNA.

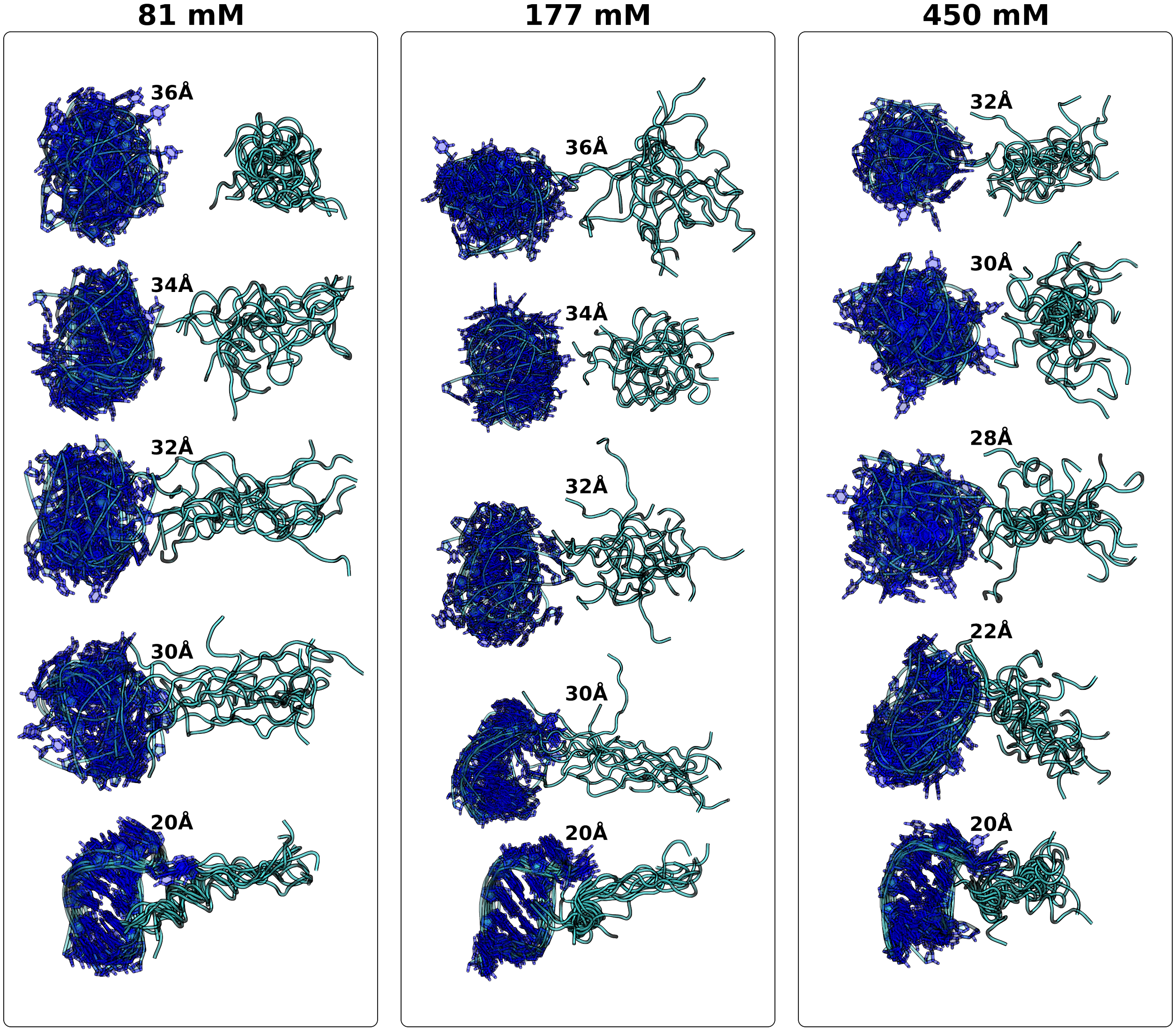

Figure S7: Visualisation of peptide orientations for different center-of-mass-distances (indicated in bold font) for the TIP4P-D water model. The peptide and RNA coordinates were taken as snapshots from the corresponding US window and were rotationally and translationally aligned with respect to the RNA-peptide center-of-mass-vector to illustrate the peptide and RNA orientation. For higher center-of-mass-Distances the orientations of the peptide (cyan cartoon) and RNA (blue cartoon) are relatively random while they become more and more defined with decreasing distances and the formation of contacts to the RNA.

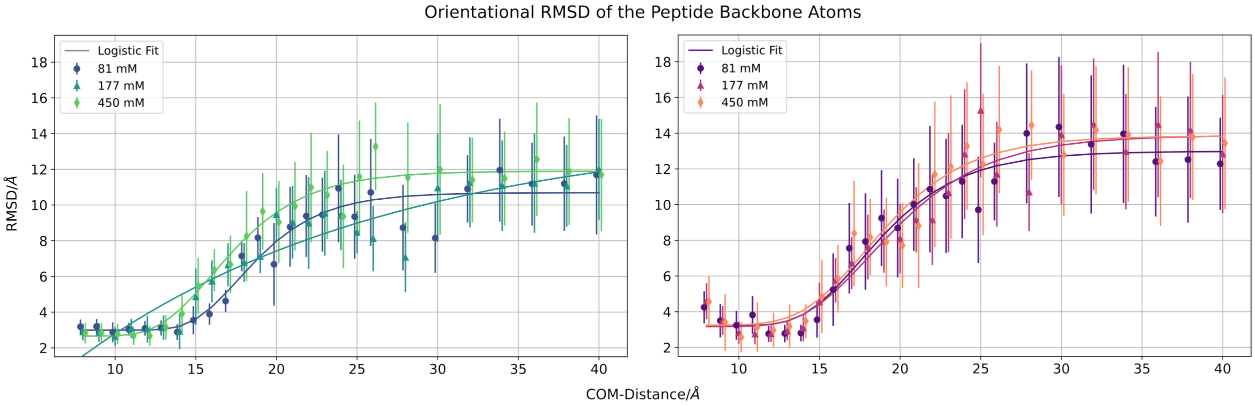

Figure S8: The orientational RMSD vs the peptide-RNA center-of-mass-distance is shown for all salt concentrations and water models (left TIP3P, right TIP4P-D). The orientational RMSD is calculated by first best-fit aligning the system with respect to the PDB RNA, followed by center-of-mass aligning the peptide to its reference and finally averaging the peptide's atomic deviations. Hence, a zero orientational RMSD means the peptide has the same orientation and structure as in the complex and a large orientational RMSD indicates a large reorientation and/or refolding relative to the experimental complex. The error bars show the standard deviation of the mean for each US window. The average is shown, if a window was simulated multiple times independently. The lines show the fitting curve of the generalised logistic function.[2]

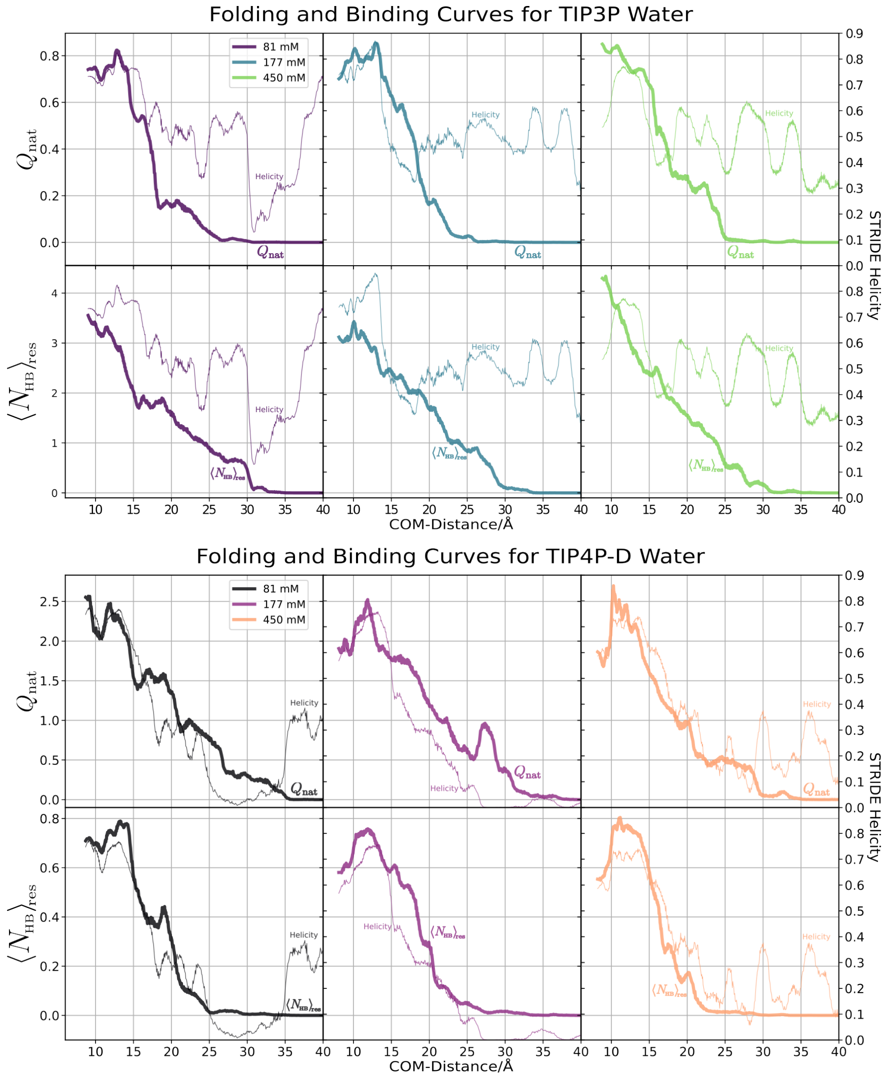

Figure S9: Native contact (Q_{nat}) and inter-molecular H-bond formation during association/dissociation of N-peptide and boxB RNA along the center-of-mass-distance coordinate. The thin line represents the calculated mean helicity.

References

(1) Klimovich, P. V.; Shirts, M. R.; Mobley, D. L. Guidelines for the analysis of free energy calculations. *Journal of Computer-Aided Molecular Design* **2015**, *29*, 397–411.

(2) Richards, J.; Lenk, E. V.; Botstein, D. Mechanism of head assembly and DNA encapsulation in Salmonella phage P22. *Journal of Molecular Biology* **1973**, *80*, 697–731.
